# Structural, Electronic, and Optical Properties of Monoclinic Pharmaceutical Crystals: A DFT Study of Salicylic Acid, Acetylsalicylic Acid, Acetaminophen, and Ibuprofen

**DOI:** 10.1021/acsomega.5c06006

**Published:** 2026-01-26

**Authors:** Liciane L. Ferreira, Mariana S. Alves, Micael E. P Oliveira, Valder N. Freire, Bruno P. Silva, José B. Silva, Ewerton W. S. Caetano

**Affiliations:** † Departamento de Física, 28121 Universidade Federal do Ceará, Caixa Postal 6030, 60440-900 Fortaleza, CE, Brazil; ‡ Computational and Quantum Chemistry Group, Instituto Federal de Educação, Ciência e Tecnologia do Ceará, Campus Camocim, 62400-000 Camocim, CE, Brazil; § Departamento de Química Biológica, 226206 Universidade Regional do Cariri, 63105-000 Crato, CE, Brazil; ∥ Departamento de Física e Matemática, Instituto Federal de Educação, Ciência e Tecnologia do Ceará, Campus Fortaleza, 60040-531 Fortaleza, CE, Brazil

## Abstract

The solid-state properties of pharmaceutical compounds play a critical role in their therapeutic efficacy, influencing their solubility, bioavailability, and stability. In this study, we investigate the monoclinic crystalline forms of four widely used anti-inflammatory drugs—salicylic acid, acetylsalicylic acid (aspirin), acetaminophen (paracetamol), and ibuprofen—using density functional theory (DFT). Employing the Perdew, Burke, and Ernzerhof (PBE) functional with Tkatchenko–Scheffler dispersion correction, we performed geometry optimizations of the unit cells, achieving lattice parameters within 1–2% of experimental values. Time-dependent DFT (TD-DFT) calculations revealed molecular UV–vis absorption spectra consistent with experimental data, elucidating key electronic transitions. Kohn-Sham band structure analyses using the HLE17 functional identified indirect band gaps ranging from 2.99 eV (salicylic acid) to 4.02 eV (ibuprofen) with near-direct transitions suggesting potential optical activity. For the acetylsalicylic acid crystal, the calculated optical absorption spectrum reproduces the main experimental features after a rigid energy shift, highlighting the effectiveness and limitations of the DFT-PBE + TS approach for describing its optical properties. Optical absorption and dielectric function calculations for light polarized along the (100), (010), and (001) crystal directions highlighted anisotropic responses tied to crystal packing and hydrogen-bonding networks. These findings provide a comprehensive understanding of the interplay among the molecular structure, crystal lattice, and optoelectronic properties, offering insights and providing a theoretical foundation for the rational design of pharmaceutical formulations with enhanced performance.

## Graphical abstract

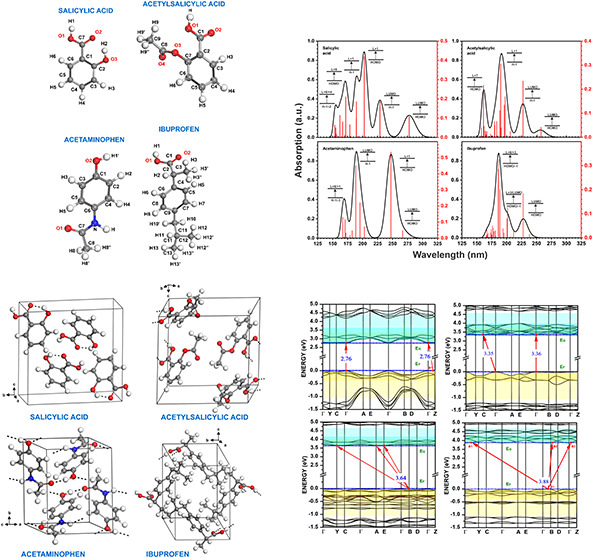

## 1 Introduction

Salicylic acid, acetylsalicylic acid (commonly known as aspirin), acetaminophen (paracetamol), and ibuprofen are foundational molecules in pharmacology and medicine, celebrated for their analgesic, antipyretic, and anti-inflammatory properties.^1,2^ These compounds, although structurally diverse, share a common thread in their widespread clinical utility, yet their efficacy and formulation are intricately tied to their solid-state properties, particularly their crystalline forms. The electronic, optical, and structural characteristics of these crystals influence critical pharmaceutical attributes, such as solubility, bioavailability, and stability under varying environmental conditions. Advances in computational techniques, notably density functional theory (DFT), have enabled detailed exploration of these properties at the atomic level, offering insights that complement experimental approaches.^3–9^

Salicylic acid, a β-hydroxy acid historically derived from willow bark (Salix alba), has been utilized for centuries as a remedy for pain and inflammation before its synthetic production became widespread.^10,11^ Chemically, it consists of a benzene ring substituted with a hydroxyl group and a carboxyl group, enabling it to form hydrogen-bonded dimers that dominate its monoclinic crystal structure (space group *P*2_1_/*c*).^3,12^ These dimers, stabilized by strong intermolecular interactions, contribute to its relatively low solubility in water (approximately 2.2 g/L at 25 °C^13^) and its photochemical stability, which are critical for its role as a topical keratolytic agent^14^ and a metabolic precursor to acetylsalicylic acid.^10^ One study has employed solid-state NMR to confirm the lattice arrangement,^15^ while vibrational spectroscopy has elucidated the dynamic interplay of hydrogen bonding and lattice phonons.^8^ Understanding its crystalline properties is essential as they influence its degradation pathways and interactions with biological systems, particularly in dermatological applications.

Acetylsalicylic acid is the acetylated derivative of salicylic acid engineered to enhance its oral bioavailability and reduce gastric irritation compared to its parent compound. Its pharmacological action stems from the irreversible inhibition of cyclooxygenase enzymes (COX-1 and COX-2), making it a versatile drug for pain relief, fever reduction, and prevention of cardiovascular events.^16–19^ Acetylsalicylic acid typically crystallizes in a monoclinic form (Form I, space group *P*2_1_/*c*),^3,20^ but a metastable polymorph (Form II) has been identified, distinguished by subtle differences in hydrogen bonding and π orbital interactions.^21–25^ Neutron diffraction and computational modeling have clarified the energetic proximity of these polymorphs, revealing how packing influences its melting point (approximately 135 °C) and solubility.^21,22,26–28^

Acetaminophen stands apart from traditional NSAIDs due to its limited anti-inflammatory activity, yet it remains a cornerstone analgesic and antipyretic, particularly valued for its favorable safety profile in vulnerable populations such as children and pregnant women.^29,30^ Structurally, it features a phenol ring with an acetamide substituent, forming extensive hydrogen-bonding networks in its crystalline phases.^31,32^ The stable Form I (monoclinic, P2_1_/n) is the commercially dominant polymorph,^33,34^ while Form II (orthorhombic) exhibits enhanced solubility, a property linked to its less dense packing.^32,35^ Neutron diffraction and molecular dynamics studies have mapped these hydrogen-bonding motifs, correlating them with its moderate solubility (20.2 g/L at 20 °C) and thermal stability.^36,37^

Ibuprofen, a propionic acid derivative, is a widely used NSAID effective against a spectrum of inflammatory conditions, from acute pain to chronic diseases like rheumatoid arthritis, due to its reversible inhibition of prostaglandin synthesis.^38^ As a racemic mixture, it adopts a monoclinic crystal structure (space group *P*2_1_/*c*), characterized by carboxylic acid dimers that dictate its low aqueous solubility (21 mg/L at 25 °C) and high lipophilicity.^39^ Recent investigations using Raman spectroscopy and DFT have probed its conformational flexibility and intermolecular interactions, revealing how crystal packing influences its melting point (75–78 °C) and dissolution kinetics.^40^ The solid-state behavior of ibuprofen is particularly relevant in formulation design, where solubility enhancements via cocrystals or amorphous forms are actively pursued to improve its therapeutic delivery.^41,42^

The study of these pharmaceuticals in the solid state is important as their electronic and optical properties underpin their stability, dissolution behavior, and interactions with light and biological environments. In this context, density functional theory (DFT) provides a powerful computational tool, offering detailed insights into molecular crystals where experimental techniques may be limited.^43–45^ For such systems, van der Waals (vdW) interactions are essential for lattice cohesion, yet standard DFT functionals such as PBE often fail to capture these forces accurately. Dispersion corrections address this limitation, improving the prediction of lattice parameters, vibrational spectra, and cohesive energies, as demonstrated in benchmark studies of molecular crystals.^46–48^ Furthermore, DFT enables the calculation of electronic band structures, optical absorption, and dielectric properties, revealing band gap characteristics and anisotropic responses to polarized light—factors linked to photochemical stability and potential optoelectronic applications.^49–51^ These capabilities make DFT an indispensable approach for bridging molecular-level understanding with macroscopic pharmaceutical properties. Our research group has been publishing several studies over the past years, successfully applying the DFT formalism with dispersion correction to molecular crystals of amino acids,^44,52–54^ nucleobases,^55^ and pharmaceuticals^56–58^ for determining their structural and optoelectronic properties.

In addition, the computational investigation of molecular crystals benefits greatly from methodologies benchmarked in the broader materials science community. For instance, the study of optoelectronic and dielectric properties in halide perovskites has seen the extensive use of density functional theory (DFT). These studies have successfully employed a range of functionals, from standard generalized gradient approximation (GGA) to modified Becke–Johnson potentials (GGA + mBJ) and hybrid functionals like HSE06, to accurately model their electronic structures and optical responses.^59–63^ The insights gained from halide perovskite research—materials also known for their significant anisotropic optical and excitonic behavior—underscore the necessity of robust theoretical approaches.^64–73^ Specifically, the challenges in accurately capturing noncovalent interactions and excited-state properties in those systems have highlighted the critical importance of incorporating dispersion corrections within DFT and employing time-dependent DFT (TD-DFT) for reliable predictions.^74–76^ Therefore, applying a similar rigorous computational strategy to anti-inflammatory molecular crystals allows us to frame their analysis within this wider context of advanced functional materials, ensuring a comprehensive understanding of their fundamental electronic and optical properties.

In this work, we employ DFT-based computational simulations to investigate the monoclinic crystalline forms of salicylic acid, acetylsalicylic acid, acetaminophen, and ibuprofen. Our methodology encompasses: (1) Time-dependent DFT (TD-DFT) calculations to compute UV–vis spectra of the crystal molecules, elucidating their electronic transitions; (2) geometry optimization of the unit cells using the PBE functional with dispersion correction, validated against experimental crystallographic data; (3) Kohn-Sham band structure calculations to determine the main band gaps and their nature (direct or indirect); and (4) optical absorption and complex dielectric function analyses for light polarized along the (001), (010), and (100) crystal directions, highlighting anisotropic optical responses. These simulations aim to provide a comprehensive understanding of the electronic and optical properties of these pharmaceutical crystals, advancing their rational design and optimization for therapeutic applications.

## 2 Methodology

Density functional theory (DFT) calculations were performed using the CASTEP code^49,51,77^ to investigate the structural, electronic, and optical properties of salicylic acid, acetylsalicylic acid, acetaminophen, and ibuprofen crystals considering experimental structures as inputs.^3,27,34,78^ The generalized gradient approximation (GGA) of Perdew, Burke, and Ernzerhof (PBE^79^) was employed for the exchange–correlation functional, complemented by the Tkatchenko–Schefler dispersion correction^80^ to account for van der Waals interactions, which are particularly relevant in molecular crystals.^81–84^ A full geometry optimization was carried out, allowing the relaxation of all unit cell parameters to ensure an accurate representation of the crystal structures in their equilibrium state. The convergence criteria for geometry optimization were chosen to ensure numerical accuracy, with a total energy variation threshold of 5 × 10^–6^ eV/atom, a maximum atomic force of 0.01 eV/Å, a maximum stress of 0.02 GPa, and an atomic displacement criterion of 5 × 10^–4^ Å. The Broyden–Fletcher–Goldfarb–Shanno (BFGS) algorithm was utilized for energy minimization due to its efficiency in handling structural relaxations of molecular systems.^85^

Self-consistent field (SCF) calculations were carried out using a plane-wave basis set with a kinetic energy cutoff of 830 eV, ensuring sufficient accuracy in the representation of Kohn-Sham orbitals while maintaining computational efficiency. Norm-conserving pseudopotentials^86^ were applied to describe core electrons, while valence electron configurations were explicitly treated as H (1s), C (2s^2^ 2p^2^), N (2s^2^ 2p^3^), and O (2s^2^ 2p^4^). The SCF cycle was deemed converged when the total energy variation between two successive iterations was below 5 × 10^–7^ eV, with a window of three consecutive iterations to ensure stability. FFT grids were tailored for each crystal to optimize computational efficiency while maintaining precision in reciprocal space integration: 48 × 108 × 108 for salicylic acid, 108 × 64 × 108 for acetylsalicylic acid, 72 × 90 × 120 for acetaminophen, and 144 × 75 × 100 for ibuprofen. The Monkhorst-Pack ***k***-point grid^87,88^ was also adapted to each system based on its unit cell dimensions and symmetry considerations, ensuring an appropriate sampling of the Brillouin zone: 3 × 1 × 1 for salicylic acid, 1 × 2 × 1 for acetylsalicylic acid, 2 × 2 × 1 for acetaminophen, and 1 × 2 × 1 for ibuprofen.

Band structure calculations were performed with an additional SCF convergence criterion, requiring an energy variation below 1 × 10^–5^ eV between successive steps to ensure precision in electronic-structure determination. Optical properties were evaluated by computing the absorption spectra and complex dielectric function, considering only electronic transitions. The calculations were performed for three distinct polarizations of incident light along the (100), (010), and (001) crystallographic directions, as well as for a polycrystalline sample to approximate the average response in an isotropic medium. These calculations provide insight into the optically anisotropic behavior of the crystals.

## 3 Structural Characteristics of Molecules and Crystals of Anti-Inflammatory Drugs

The structures of the four investigated molecules, along with their respective atom nomenclatures, are shown in Figure 1, while Figure 2 presents the unit cells of their monoclinic crystals. Salicylic acid (2-hydroxybenzoic acid) is constituted by a benzene core substituted at the C1 position with a carboxyl (–COOH) group and with a hydroxyl (–OH) group at the C2 position, forming an ortho-hydroxybenzoic acid framework with a planar conformation exhibiting minor distortions due to electronic effects and intermolecular interactions. The intramolecular O–H···O hydrogen bond between the hydroxyl and carboxyl groups stabilizes the structure, reinforcing the coplanarity of these functional groups with the benzene ring and contributing to the acid strength of the molecule. The benzene ring retains an almost ideal hexagonal geometry. The monoclinic crystal unit cell of salicylic acid, determined via X-ray diffraction,^3,89,90^ reveals two dimeric arrangements mostly due to the formation of O–H···O hydrogen bonds, supplemented by weaker C–H···O and π···π interactions, stabilizing the overall crystal lattice. The space group is *P*2_1_/*c*, with unit cell parameters of *a* = 4.88 Å, *b* = 11.20 Å, *c* = 11.42 Å, and β = 92.62°. The molecular geometry in the crystal is also influenced by resonance effects, resulting in partial double-bond character in C–C and C–O bonds, with measured lengths deviating slightly from ideal single and double bonds. Electron density mapping shows a small but significant departure from spherical symmetry, with localized electron density enhancements along covalent bonds, suggesting charge delocalization. Thermal vibration analysis indicates anisotropic atomic motion, particularly in oxygen atoms, perpendicular to the C–O bonds.^89^

**Figure 1:**
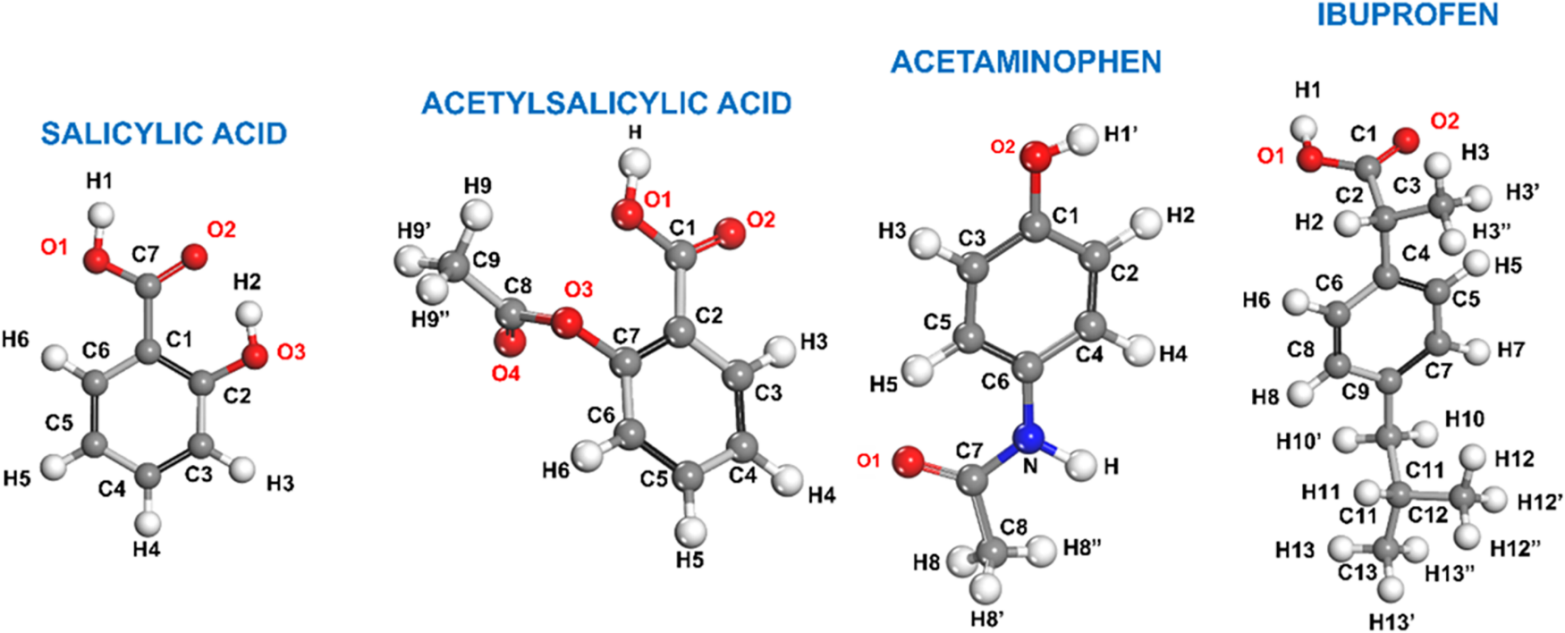
Molecules of the four anti-inflammatory drugs under study with their respective atom labels.

**Figure 2:**
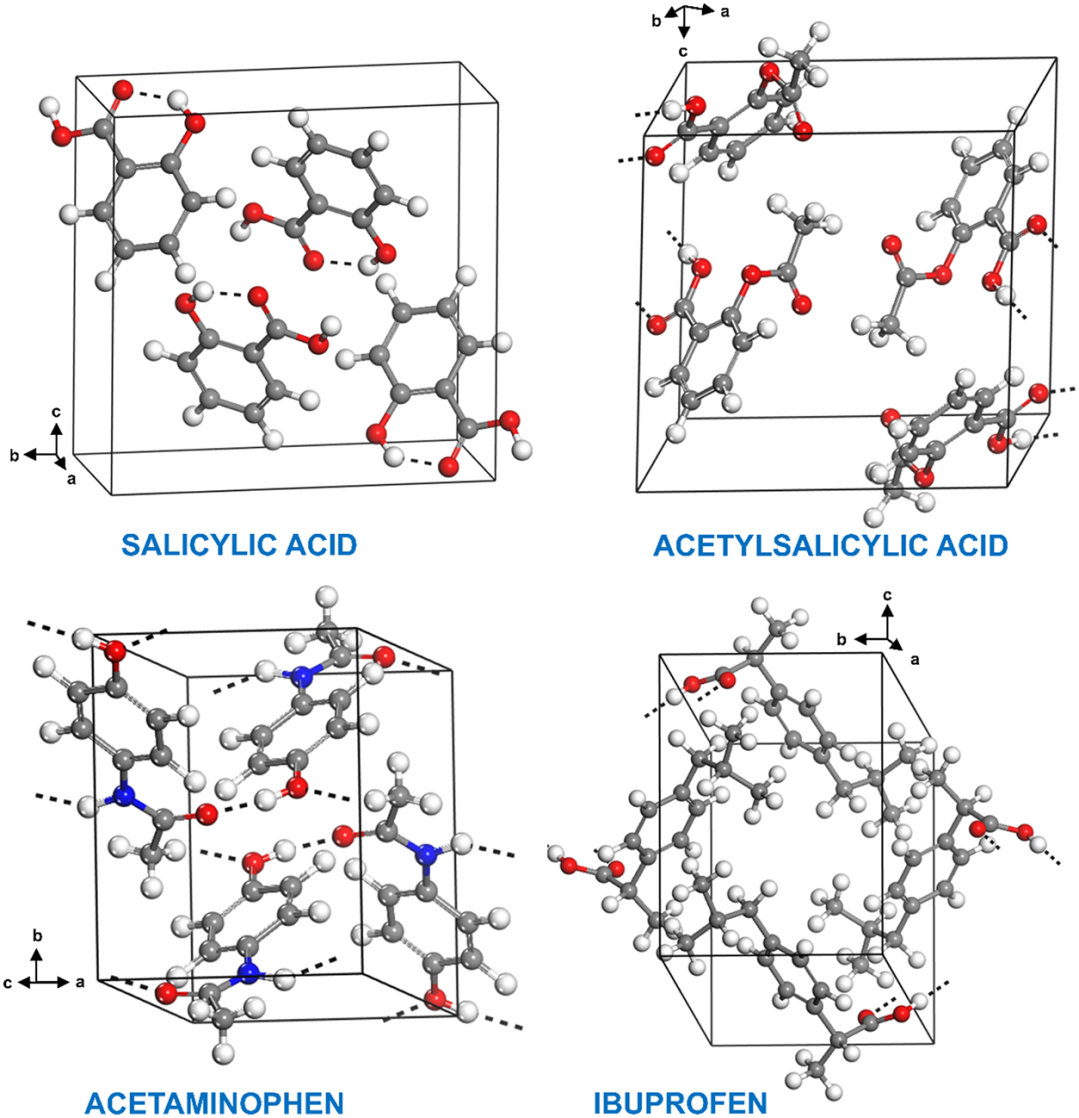
Unit cells of monoclinic crystals of salicylic acid, acetylsalicylic acid, acetaminophen, and ibuprofen. Hydrogen bonds are shown as dashed lines.

Compared to salicylic acid, the molecular structure of acetylsalicylic acid (2-acetoxybenzoic acid) exhibits distinct electronic and steric effects due to the substitution of the hydroxyl (–OH) group with an acetoxy (–OCOCH_3_) group at the C2 position. While the intramolecular O–H···O hydrogen bond between the hydroxyl and carboxyl groups in salicylic acid stabilizes a nearly planar conformation, the ester group in acetylsalicylic acid introduces additional steric hindrance, slightly distorting the molecular planarity. The carboxyl functional group in both molecules exhibits C=O and C–O bond lengths indicative of resonance delocalization. However, in acetylsalicylic acid, the absence of the hydroxyl proton eliminates the strong intramolecular hydrogen bond, modifying the electronic environment around the carboxyl group. The benzene ring remains structurally similar in both molecules, with C–C bond lengths characteristic of aromatic delocalization. While both molecules form O–H···O hydrogen-bonded dimers in their crystal unit cells, acetylsalicylic acid exhibits weaker hydrogen bonding and interactions of the bulkier acetoxy group with π orbitals of the aromatic ring, as well as π···π interactions, leading to differences in molecular packing and crystal stability.^20^ The crystal structure of acetylsalicylic acid, as determined by variable-temperature single-crystal neutron diffraction,^27^ reveals a monoclinic *P*2_1_/*c* space group with four molecules per unit cell. The structure consists of centrosymmetric dimers in which two molecules are linked via a carboxylic acid dimerization motif, forming two hydrogen bonds. Unlike salicylic acid, the acetoxy (–OCOCH_3_) group is oriented nearly perpendicular to the benzene ring, with a C1–C2–O3–C8 torsion angle of ∼81.8° at 100 K, minimizing steric interactions.

The molecular structure of acetaminophen (*N*-acetyl-4-aminophenol, C_8_H_9_NO_2_) consists of a benzene ring substituted at the C1 position with a hydroxyl (–OH) group and with an acetamide (–NHCOCH_3_) group at the C4 position, forming a 4-hydroxyacetanilide framework. The molecule adopts a planar conformation with minor deviations due to intramolecular hydrogen bonding and steric effects. The acetamide functional group exhibits C=O and C–N bond lengths consistent with partial resonance delocalization, leading to restricted rotation around the C–N bond. The hydroxyl group is involved in strong intramolecular hydrogen bonding with the adjacent amide oxygen, influencing the electronic distribution within the molecule. The benzene ring retains an aromatic character with C–C bond lengths typical of a delocalized π-electron system. Electron density mapping highlights the anisotropic charge distribution at the oxygen and nitrogen sites, reinforcing the ability of the molecule to form extensive intermolecular hydrogen bonds in the solid state. In the crystalline form, acetaminophen molecules exhibit O–H···O and N–H···O hydrogen bonding, creating a three-dimensional network of molecular interactions that significantly impact its physicochemical properties, solubility, and stability.^34^ Its monoclinic unit cell belongs to space group *P*2_1_/*n*. The benzene has carbon atom deviations from the mean plane not exceeding 0.007 Å, reflecting its structural stability, while the acetamide (hydroxy) group forms a dihedral angle of 20.5° (17.2°) with the benzene ring. Intermolecularly, each molecule engages with six neighbors through two hydrogen bonds: O–H···O=C and N–H···O–H, organizing into pleated sheets parallel to the (101) plane within the monoclinic lattice. These sheets stack along the [010] direction via van der Waals interactions, and form “head-to-tail” dimers between sheets, with an N1-to-benzene ring centroid separation of 3.33 Å. Hydrogen-bonded chains along [100] align with anisotropic properties like sublimation and dissolution, emphasizing the influence of the *P*2_1_/*n* space group and monoclinic unit cell on the molecular arrangement and crystal packing of this pharmaceutical compound.

Lastly, ibuprofen (C_13_H_18_O_2_, α-methyl-4-(isobutyl)­phenylacetic acid) is formed from a phenyl ring substituted at the para position with an isobutyl (–CH_2_CH­(CH_3_)_2_) group and at the α position with a carboxyl (–COOH) group, creating a 2-(4-isobutylphenyl)­propionic acid framework. Its molecule adopts a nonplanar conformation, primarily due to the steric effects of the bulky isobutyl and carboxyl groups. The phenyl ring remains nearly planar, with C–C bond lengths reflecting delocalized π-electron density.^40,91^ The presence of the asymmetric α-methyl group results in chiral properties, with ibuprofen existing as (S)- and (R)-enantiomers, where the (S)-form is biologically active as a nonsteroidal anti-inflammatory drug (NSAID). In the solid state, it has a monoclinic unit cell with a *P*2_1_/*c* space group. Intermolecular O–H···O hydrogen bonding between carboxyl groups stabilizes the crystal packing, supplemented by van der Waals interactions between alkyl chains. Crystallographic studies^78,92,93^ reveal that the benzene ring maintains planarity. The carboxyl group of propionic acid facilitates hydrogen bonding in the solid state, often forming centrosymmetric dimers as commonly observed in carboxylic acid-containing compounds. The isobutyl group, with its branched aliphatic structure, extends outward, contributing to hydrophobic interactions and influencing the molecular packing within the crystal lattice, typically aligning molecules into chains or layers stabilized by van der Waals forces.

## 4 Results

### 4.1 TD-DFT Molecular Simulation

Molecular geometry optimizations were performed for each anti-inflammatory molecule using Gaussian 16.^94^ The hybrid functional HSE06 ^95^ was employed in combination with the Pople basis set 6-311++G­(2d,2p) for density functional theory (DFT) and time-dependent DFT (TD-DFT) calculations. Geometry optimizations were carried out using a convergence threshold of 2 × 10^–6^ Ha/Å for the maximum force, with an RMS value of 10^–6^ Ha/Å, and a maximum displacement of 6 × 10^–6^ Å with an RMS of 4 × 10^–6^ Å. The minimum energy configuration was confirmed by vibrational frequency calculations, ensuring the absence of imaginary frequencies. To account for solvent effects, the polarizable continuum model (PCM^96^) was applied. Water (ε = 78.39) was chosen as the implicit solvent for all of the molecules. This selection provides a standardized, highly polar environment, enabling a consistent comparison of the intrinsic electronic properties across different systems. Furthermore, this choice facilitates comparison with a broad range of experimental UV–vis spectra reported in the literature, which are often measured in aqueous solutions.^97–101^ We note that the use of different formulation-specific solvents would likely lead to solvatochromic shifts in the absorption maxima.^102,103^ However, a detailed investigation of such solvent-dependent effects was considered beyond the scope of this foundational study, but it represents an interesting path for future application-oriented research.

Self-consistent field (SCF) calculations were performed with a convergence criterion of 1 × 10^–10^ Ha. After geometry optimization, electronic-structure calculations were conducted within the TD-DFT framework. The absorption spectra were analyzed using the auxiliary program GaussSum,^104^ identifying the molecular orbitals contributing most significantly to the observed UV–vis absorption peaks.

The frontier molecular orbitals (FMOs), namely, the highest occupied molecular orbital (HOMO) and the lowest unoccupied molecular orbital (LUMO), were computed for the four anti-inflammatory drugs and are depicted in Figure 3. For all studied compounds—salicylic acid, acetylsalicylic acid, ibuprofen, and acetaminophen—the FMOs are predominantly of π character, delocalized over their respective aromatic ring systems. Consequently, the lowest-energy electronic transition is characterized as a π → π* excitation. This transition involves the promotion of an electron from the bonding π orbital (HOMO) to the corresponding antibonding π* orbital (LUMO), which is fundamental to the UV absorption properties of these molecules.

**Figure 3:**
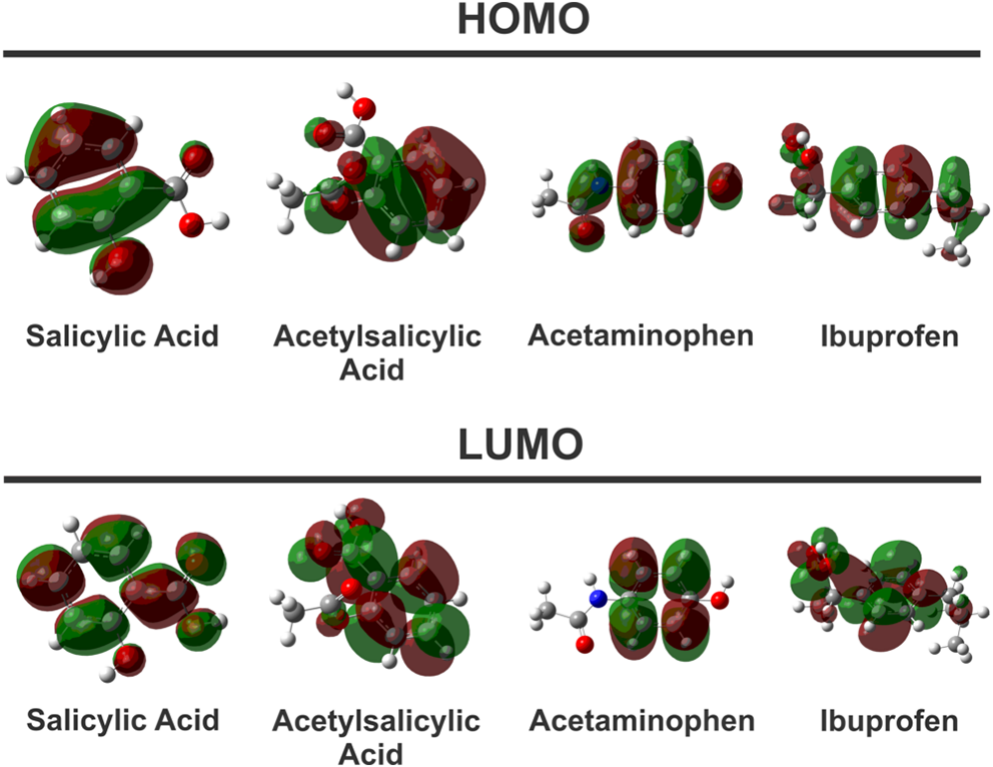
Frontier orbitals (HOMO and LUMO) were obtained for the four anti-inflammatory molecules using the TD-DFT method.

The spatial distribution of the FMOs underscores the differentiated electronic influence exerted by each substituent. In salicylic acid, the HOMO exhibits delocalization over the phenyl ring, hydroxyl, and carboxyl functionalities, whereas the LUMO is predominantly localized on the phenyl ring and carboxyl group, with a negligible contribution from the hydroxyl oxygen. In acetylsalicylic acid, a comparable delocalization pattern is observed, encompassing the aromatic ring together with the carboxyl and ester moieties. In acetaminophen, HOMO is extensively delocalized across the conjugated framework, incorporating the phenyl ring, as well as the heteroatoms of the amide and hydroxyl substituents. By contrast, the FMOs of ibuprofen remain largely confined to the aromatic fragment: while HOMO extends over the entire molecular framework, LUMO systematically avoids the saturated aliphatic chain, evidencing pronounced electronic decoupling of the side chain from the π-conjugated system.

Figure 4 presents the absorption spectra and main electronic transitions of salicylic acid, acetylsalicylic acid, ibuprofen, and acetaminophen molecules in aqueous solution. For salicylic acid, our results agree with the study by Farouq and Selim,^105^ which reported an absorption peak around 300 nm. Trivedi et al.^106^ also observed this peak at 302.4 nm, along with two additional absorption peaks at 234.4 and 206.8 nm. In our TD-DFT calculations at the HSE06/6-311++G­(2d,2p) level, the first peak appears at 278 nm, corresponding to the HOMO → LUMO transition, while the other two are located at 229.18 nm (H – 1 → LUMO) and 203.04 nm (HOMO → L + 1), consistent with the findings of Trivedi et al.

**Figure 4:**
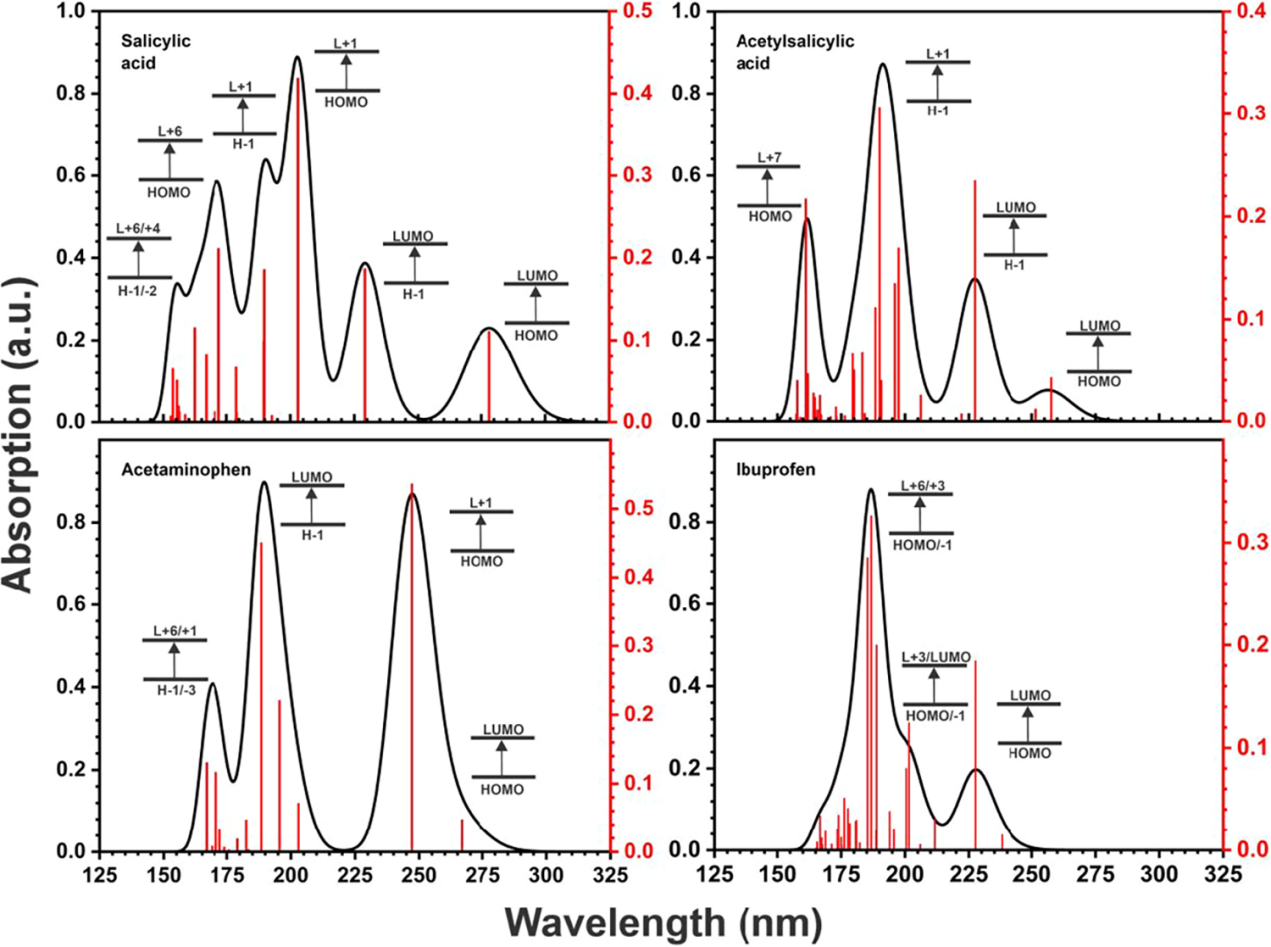
Optical absorption spectra calculated using TD-DFT simulations for the molecules of salicylic acid, acetylsalicylic acid, acetaminophen, and ibuprofen. The electronic transitions corresponding to the most significant peaks are indicated.

The UV–vis spectrum of acetylsalicylic acid exhibits good agreement with experimental data reported in the literature,^107,108^ which identify two absorption peaks around 220 and 300 nm. Our theoretical results indicate that the most significant electronic transitions in this spectral region occur at 229.17 nm (H – 1 → LUMO) and 278.07 nm (HOMO → LUMO). The UV–vis spectrum of acetaminophen is consistent with prior experimental work,^109^ which reported two absorption peaks. However, due to the full width at half-maximum (fwhm) values used in our calculations, these peaks overlap, resulting in a combined absorption feature localized at 247.20 nm (HOMO → L + 1) and 266.75 nm (HOMO → LUMO). Finally, for ibuprofen, Marbán et al.^110^ reported two absorption peaks: a strong peak in the 190–200 nm region and a less intense peak in the 220–330 nm range. Our calculations suggest that the primary transitions responsible for the first peak are HOMO → L + 3 and H – 1 → LUMO, whereas for the second peak, the dominant contribution comes from the HOMO → LUMO transition.

### 4.2 Crystal Geometry Optimizations

Table 1 shows the unit cell parameters for the four anti-inflammatory drug crystals evaluated using the GGA-PBE functional enhanced by the Tkatchenko–Scheffler (TS) dispersion correction at plane-wave cutoff energy of 830 eV (additional structural data are available in the Supporting Information). The theoretical lattice parameters (*a, b, c*), unit cell volumes (*V*), and monoclinic angles (β) were compared against experimental measurements, with deviations (Δ*a*, Δ*b*, Δ*c*, Δ*V*, Δβ) calculated to assess the performance of the method. Overall, the computational approach yields lattice parameters and unit cell volumes that align closely with experimental values, with deviations typically within 1–2%. This level of agreement is commendable for DFT simulations of molecular crystals, where accurately balancing hydrogen bonding and dispersion interactions poses a significant challenge. However, specific discrepancies, particularly in the *c*-axis, unit cell volumes, and β angles, reveal limitations in the ability of the GGA-PBE functional to fully capture the anisotropic nature of intermolecular forces, even with the TS dispersion correction.

**Table 1:** Lattice Parameters *a*, *b*, *c*, *V*, β, and Their Deviations Δ*a*, Δ*b*, Δ*c*, Δ*V*, and Δβ from Experimental Values for the Unit Cells of Anti-inflammatory Crystals

	*a* (Å)	Δ*a* (Å)	*b* (Å)	Δ*b* (Å)	*c* (Å)	Δ*c* (Å)	*V* (Å^3^)	Δ*V* (Å^3^)	β (deg)	Δβ (deg)
Salicylic Acid										
Exp^3^	4.88		11.20		11.23		613.61		92.62	
GGA + TS	4.87	–0.01	11.20	0.00	11.42	+0.19	622.76	+9.14	91.43	–1.19
Acetylsalicylic Acid										
Exp^27^	11.23		6.54		11.23		821.22		95.89	
GGA + TS	11.28	0.05	6.51	–0.03	11.37	0.14	828.96	7.74	97.16	1.27
Acetominophen										
Exp^34^	7.09		9.26		11.66		759.09		97.67	
GGA + TS	7.04	–0.05	9.14	–0.12	11.67	0.01	742.24	–16.85	98.63	0.96
Ibuprofen										
Exp^78^	14.40		7.82		10.51		1165.60		99.70	
GGA + TS	14.54	0.14	7.74	–0.08	10.34	–0.17	1143.73	–21.87	100.68	0.98

For salicylic acid, the experimental lattice parameters are *a* = 4.88 Å, *b* = 11.20 Å, *c* = 11.23 Å, *V* = 613.61 Å^3^, and β = 92.62°, while the GGA+TS results are *a* = 4.87 Å, *b* = 11.20 Å, *c* = 11.42 Å, *V* = 622.76 Å^3^, and β = 91.143°. The deviations are minimal for *a* and *b* (Δ*a* = −0.01 Å, Δ*b* = −0.00 Å), but more pronounced for *c* (Δ*c* = +0.19 Å), resulting in a volume increase of 9.14 Å^3^ and a β deviation of −1.19°. The overestimation of the *c*-axis and underestimation of β indicate that the GGA-PBE functional, with TS correction, may result in an overestimation of the lattice expansion along directions influenced by weaker interlayer interactions. This could stem from an incomplete description of anisotropic van der Waals forces, which compete with hydrogen bonding and π–π interactions in stabilizing the structure of salicylic acid. In acetylsalicylic acid, on the other hand, experimental lattice values are *a* = 11.23 Å, *b* = 6.54 Å, *c* = 11.23 Å, *V* = 821.22 Å^3^, and β = 95.889°, compared to theoretical values of *a* = 11.28 Å, *b* = 6.51 Å, *c* = 11.37 Å, *V* = 828.96 Å^3^, and β = 97.16°. The deviations (Δ*a* = +0.05 Å, Δ*b* = −0.03 Å, Δ*c* = +0.14 Å, Δ*V* = +7.74 Å^3^, Δβ = +1.27°) indicate a slight anisotropic expansion, with *a* and *c* overestimated and *b* underestimated. The increase in β suggests an overcompensation for dispersion interactions along the *b*-axis, where hydrogen bonding is critical. This discrepancy underscores the difficulty in balancing dispersion and hydrogen-bonding effects, as the TS correction may overly enhance van der Waals contributions in directions where hydrogen bonds dominate.

In the case of acetaminophen, the experimental lattice parameters are *a* = 7.09 Å, *b* = 9.26 Å, *c* = 11.66 Å, *V* = 759.09 Å^3^, and β = 97.67°, while the computed values are *a* = 7.04 Å, *b* = 9.14 Å, *c* = 11.67 Å, *V* = 742.24 Å^3^, and β = 98.63°. The deviations (Δ*a* = −0.06 Å, Δ*b* = −0.12 Å, Δ*c* = +0.01 Å, Δ*V* = −16.85 Å^3^, Δβ = +0.96°) highlight a volume underestimation of 16.85 Å^3^, the largest relative value among the four compounds (>2.2%). This likely arises from a limited representation of the hydrogen-bonding network, which is pivotal in the crystal lattice of acetaminophen. The slight overestimation of β suggests that the GGA+TS method may exaggerate monoclinic distortion, possibly due to an overcorrection of dispersion forces along the *c*-axis, where intermolecular interactions are less prominent.

Ibuprofen presents experimental values of *a* = 14.40 Å, *b* = 7.82 Å, *c* = 10.51 Å, *V* = 1165.60 Å^3^, and β = 99.70°, against theoretical values of *a* = 14.54 Å, *b* = 7.74 Å, *c* = 10.34 Å, *V* = 1143.73 Å^3^, and β = 100.68°. The deviations (Δ*a* = +0.15 Å, Δ*b* = −0.08 Å, Δ*c* = −0.17 Å, Δ*V* = −21.87 Å^3^, Δβ = +0.98°) reveal the largest volume underestimation of 21.87 Å^3^ (−1.88%), attributable to the complex interplay of dispersion forces and weak hydrogen bonding. The GGA-PBE functional with TS correction appears to struggle with these interactions, causing a contraction along the *c*-axis and an expansion along the *a*-axis. The overestimated β further indicates an overcorrection of dispersion effects, amplifying the monoclinic angle. All of the observed discrepancies can be traced to computational limitations. The GGA-PBE functional is known to underperform in describing dispersion interactions, and while the TS correction mitigates this, it may not fully address the anisotropy of van der Waals forces in molecular crystals. The 830 eV cutoff energy ensures convergence, but subtle errors may persist due to insufficient optimization of the basis set or ***k***-point sampling. Moreover, the TS method may not adequately capture systems with intricate hydrogen bonding, as seen in acetaminophen and ibuprofen. To enhance accuracy, hybrid functionals could be employed, which better handle exchange–correlation effects (but with much higher computational cost) or more advanced dispersion corrections like the many-body dispersion (MBD) method, which accounts for collective van der Waals interactions. Temperature-dependent calculations or zero-point energy corrections could also address thermal expansion effects, although these are likely minor given the experimental context.

A summary of the structural data calculated for the hydrogen bonds in the anti-inflammatory crystals is shown in Table 2. The calculated donor (D)–acceptor (A) distances (D···A) are generally consistent with experimental values, and the calculated bond angles (D–H···A) successfully reproduce the linearity of the observed interactions. For example, the strong carboxylic acid dimer interaction in acetylsalicylic acid is calculated to have a D···A distance of 2.579 Å and an angle of 175.2°, compared to the experimental values of 2.635 Å and 177.7°, respectively. This strong correlation validates our computational model and confirms that these geometric descriptors provide a reliable basis for analyzing the role of hydrogen bonding in these systems.

**Table 2:** Hydrogen Bond Parameters of Four Anti-inflammatory Crystals: Experimental Data in Comparison with the Results of DFT Simulations

	D–H bond length (Å)		H···A distance (Å)		D···A distance (Å)		D–H···A angle (degrees)	
Salicylic Acid								
atoms involved	O1–H6	O2–H5	H6···O3	H5···O3	O2···O3	O1···O3	O1–H6···O3	O2–H5···O3
Exp	1.02504	0.963984	1.62667	1.78525	2.61914	2.64912	174.805	142.852
DFT	1.02404	0.996205	1.56243	1.67946	2.57949	2.58480	175.794	148.098

Acetylsalicylic Acid				
atoms involved	O1–H8	H8···O2	O1···O2	O1–H8···O2
Exp	1.00944	1.62656	2.63549	177.696
DFT	1.02865	1.55239	2.57885	175.182

Acetaminophen								
atoms involved	O1–H1	N1–H6	H1···O2	H6···O1	O1···O2	N1···O1	O1–H1···O2	N1–N6···O1
Exp	0.922323	0.924647	1.75237	2.01379	2.65606	2.91338	165.758	163.867
DFT	1.00390	1.02974	1.62877	1.89417	2.60487	2.90124	162.838	165.051

Ibuprofen				
atoms involved	O1–H1	H1···O2	O1···O2	O1–H1···O2
Exp	0.963064	1.66346	2.62650	179.493
DFT	1.02648	1.55822	2.58432	177.986

This geometric analysis also highlights the presence of strong, highly directional H-bonds that dictate the crystal packing in these materials. The robust intramolecular H-bonds in salicylic acid and the classic centrosymmetric carboxylic acid dimers in acetylsalicylic acid and ibuprofen are characterized by short D···A distances (all ∼2.6 Å) and nearly linear angles (>174°). Acetaminophen features a more complex three-dimensional network of O–H···O and N–H···O interactions that connect the molecules in the crystal. The prevalence of these highly ordered and directional intermolecular forces is fundamental to the formation of the crystalline lattice. Therefore, a precise characterization of their geometry is critical to understanding the origins of macroscopic anisotropic properties, directly supporting our interpretation of the role of H-bonds in the observed optical anisotropy.

### 4.3 Electronic Band Structures

Figure 5 shows the first Brillouin zones for the crystals of anti-inflammatory drugs. Each diagram presents the reciprocal unit cell, highlighting high-symmetry points and the selected paths for electronic band structure calculations. The high-symmetry points and their respective fractional coordinates in reciprocal space are *A* (−0.5, 0.5, 0.0), *B* (−0.5, 0.0, 0.0), *C* (0.0, 0.5, 0.5), *D* (−0.5, 0.0, 0.5), *E* (−0.5, 0.5, 0.5), *Y* (0.0, 0.5, 0.0), and *Z* (0.0, 0.0, 0.5) and define the key directions along which electronic band dispersion can be analyzed. The shape and orientation of each Brillouin zone vary according to the specific crystallographic structure of each molecule, influencing the electronic properties and potential anisotropies in charge transport. The selected ***k***-paths connect these high-symmetry points to capture the most relevant features of the band structures.

**Figure 5:**
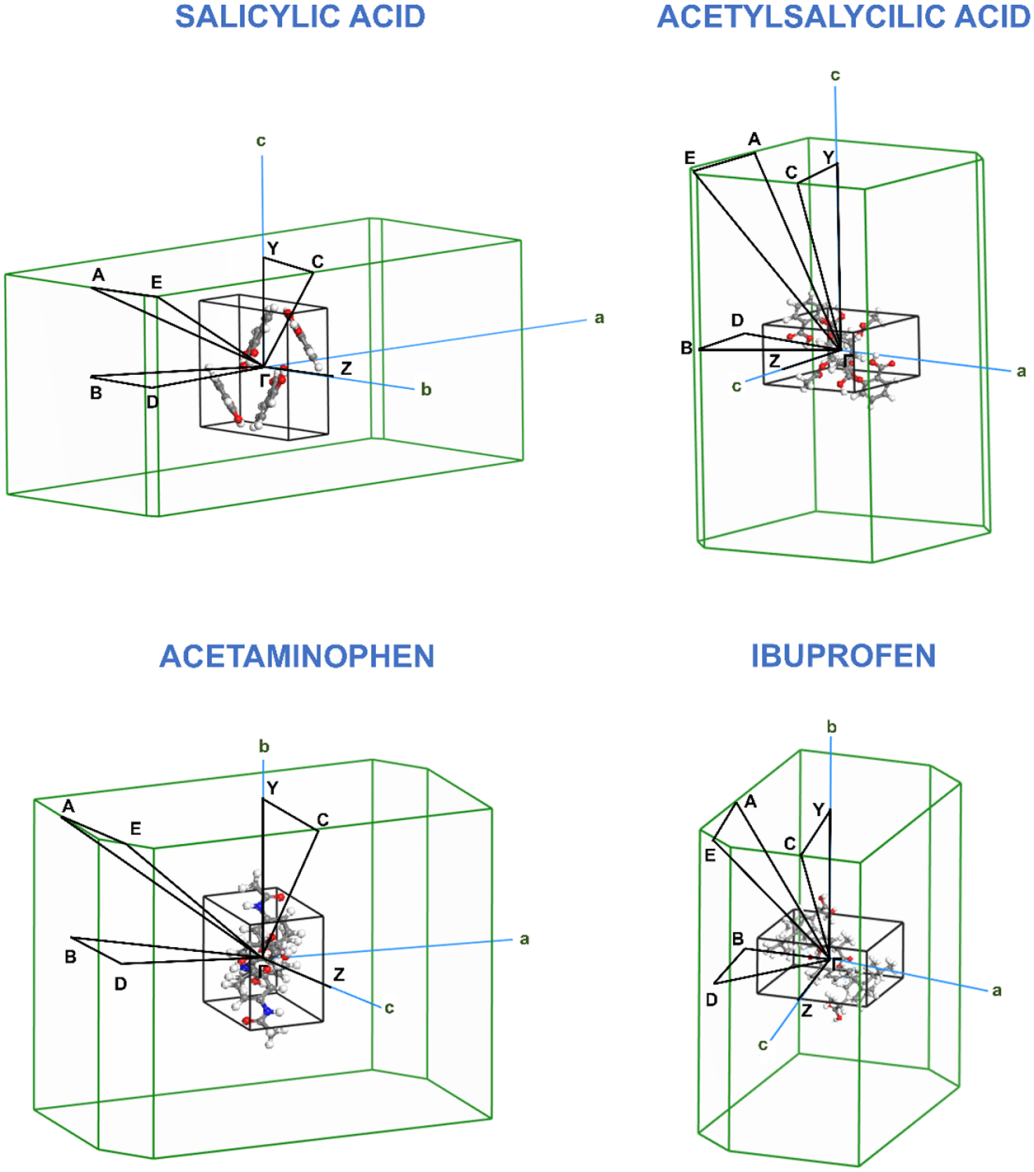
First Brillouin zones of the anti-inflammatory drug crystals.

Figures 6 and 7 present the calculated Kohn-Sham electronic band structures and partial densities of states (PDOS) of the systems under study. The plotted energy bands are referenced to the Fermi level *E*_F_ = 0 eV, with occupied states shaded in brown and unoccupied states shaded in blue. The calculated fundamental band gaps are indicated with red arrows. Salicylic acid has an indirect band gap of 2.76 eV, with the valence band maximum (VBM) located along the Γ–*Z* direction and the conduction band minimum (CBM) at Γ. It also presents a nearly identical direct Γ → Γ gap, suggesting that optical transitions at this point could be significant. The conduction bands of salicylic acid are notably flat along the Γ–Y–C–Γ path, while they display greater dispersion along the remaining paths in the Brillouin zone, revealing anisotropic electronic transport properties. Acetylsalicylic acid, on the other hand, has an indirect band gap of 3.64 eV, with both the valence and conduction bands exhibiting remarkable flatness throughout the Brillouin zone. This suggests localized electronic states for both electrons and holes, potentially leading to poor charge carrier mobility.

**Figure 6:**
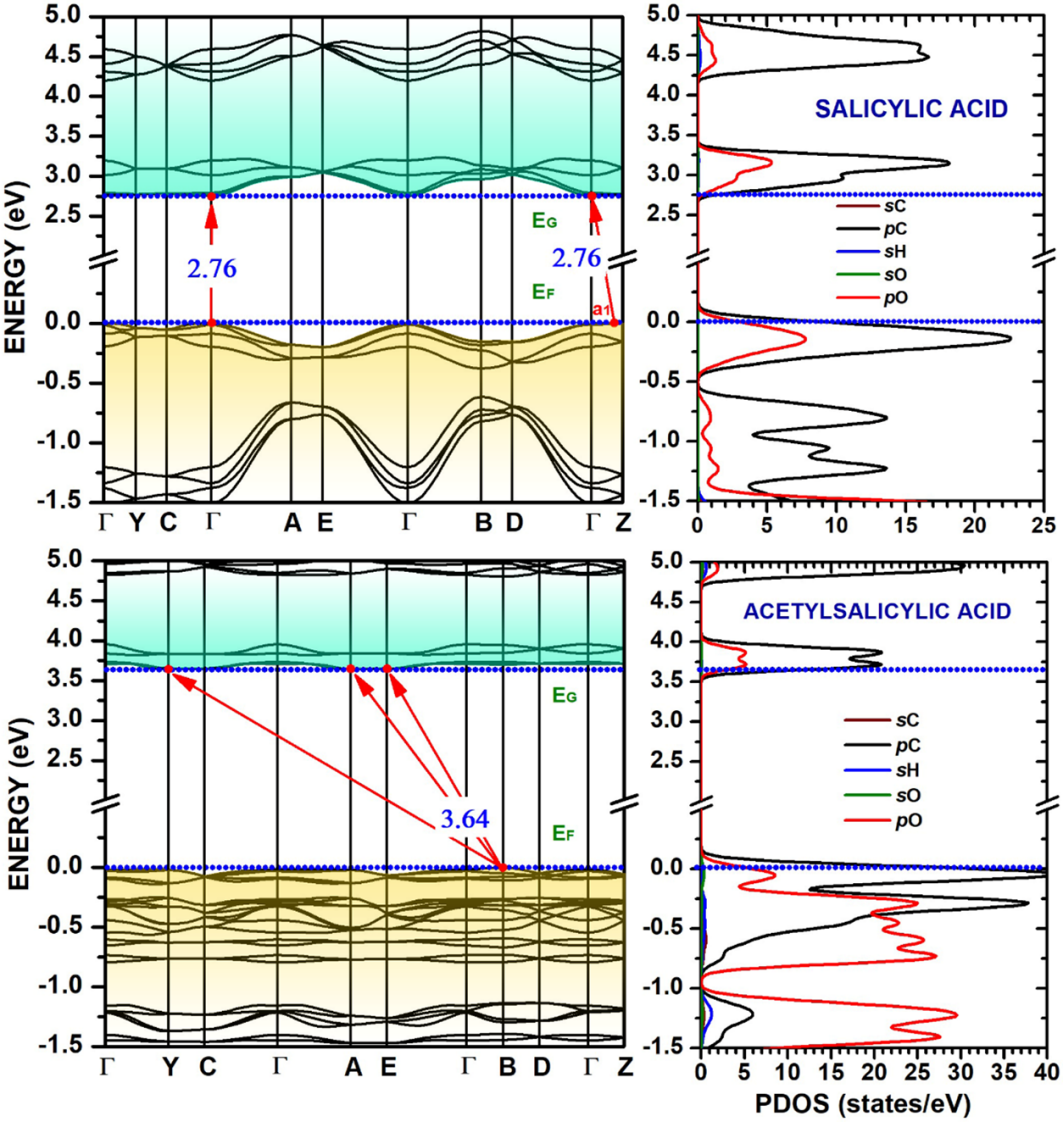
Kohn-Sham band structure (left) and partial density of states (PDOS, right) for salicylic and acetylsalicylic acid crystals. The Fermi level (*E*_F_) is set to 0 eV. Red arrows indicate the most important band gaps (*E*_g_). The PDOS panel decomposes the total DOS into contributions from s and p orbitals of carbon (sC, pC), hydrogen (sH), and oxygen (sO, pO), showing the p-orbital dominance near the band edges.

**Figure 7:**
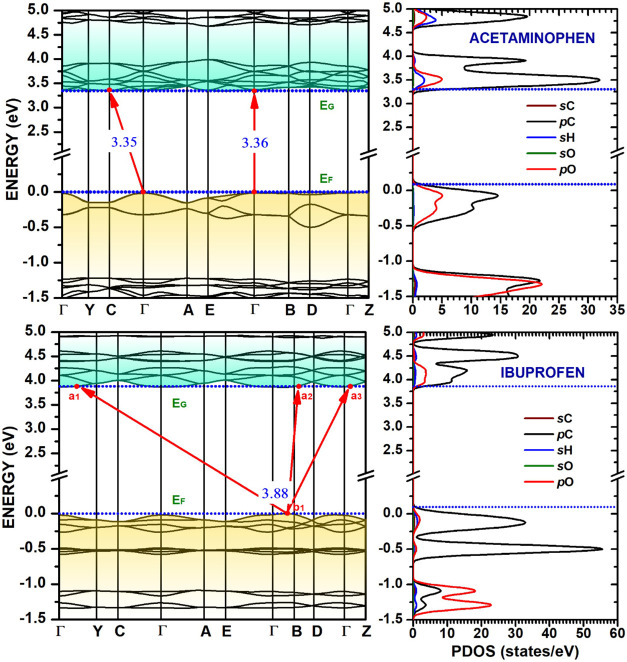
Kohn-Sham band structure (left) and partial density of states (PDOS, right) for acetaminophen and ibuprofen crystals. The Fermi level (*E*_F_) is set to 0 eV. Red arrows indicate the most important band gaps (*E*_g_). The PDOS panel decomposes the total DOS into contributions from s and p orbitals of carbon (sC, pC), hydrogen (sH), and oxygen (sO, pO), showing the p-orbital dominance near the band edges.

Acetaminophen presents an indirect band gap of approximately 3.35 eV, where the VBM is located at Γ, and the CBM lies along the Γ–*E*. Notably, it also features a direct Γ–Γ band gap that is very close in value to the indirect gap, implying that optical transitions at Γ could play a significant role in its electronic properties. Furthermore, the valence band of acetaminophen is particularly flat along the Γ–*B*–*D*–Γ–*Z* path. Ibuprofen exhibits the largest band gap among the four compounds, with an indirect gap of 3.86 eV between the VBM at *B* and the CBM along the *B*–Γ direction. Like acetaminophen, it also has a direct gap at Γ that is very close in energy to the indirect gap, potentially impacting its optical absorption properties. In contrast to the other compounds, the valence and conduction bands of ibuprofen display greater dispersion along various directions in the Brillouin zone.

It is well-established that standard density functional theory (DFT), particularly with semilocal functionals such as GGA-PBE, systematically underestimates band gaps due to its inadequate treatment of exchange interactions and lack of quasiparticle corrections. More accurate estimates can be obtained from beyond-DFT methods such as many-body perturbation theory (GW) or hybrid functionals (e.g., HSE06), but these approaches are computationally expensive, especially for systems with large unit cells such as the molecular crystals investigated here. To achieve improved band gap predictions at moderate computational cost, we employed the HLE17 meta-GGA functional. HLE17 has been shown to yield band gap values close to those of hybrid functionals such as HSE06 while requiring substantially less computational effort.^111–113^ It represents a refinement of the HLE16 GGA functional, itself recognized for reliable band gap estimations.^114^ Although hybrid functionals (e.g., HSE06 or PBE0) typically provide a more rigorous description of nonlocal exchange–correlation effects (which can be relevant in molecular crystals), the HLE17 functional offers a practical balance between accuracy and efficiency. Hybrid functionals would likely yield slightly larger band gaps and a more accurate band dispersion as well as an improved basis for dielectric function calculations. Nevertheless, considering the size of the unit cells in this study, HLE17 provides a suitable compromise between accuracy and computational feasibility for the present comparative analysis.

Our calculations show that the band gaps for salicylic acid, acetylsalicylic acid, acetaminophen, and ibuprofen increased from 2.76, 3.64, 3.37, and 3.86 eV (at the GGA-PBE level) to 2.99, 3.76, 3.56, and 4.02 eV with the HLE17 functional, respectively. Since the overall band dispersion was not significantly altered by the choice of the functional, the physical implications remain similar. The presence of nearly direct band gaps in salicylic acid, acetaminophen, and ibuprofen suggests that these materials could exhibit significant optical absorption in the UV region. The pronounced flatness of the valence and conduction bands in acetylsalicylic acid and acetaminophen implies low carrier mobilities, which could limit their performance in charge transport applications but may enhance exciton binding energies. Conversely, the greater dispersion observed in the bands of ibuprofen suggests comparatively improved carrier transport, which may be relevant for electronic applications.

The calculated partial densities of states (PDOS) near the main gap for the four anti-inflammatory drug crystals are shown at the right-side panels of Figures 6 and 7. The analysis of the orbital contributions reveals that the valence band maximum (VBM) is primarily composed of hybridized states from the 2p orbitals of carbon (pC) and oxygen (pO). However, the specific structure of the VBM differs significantly among the compounds. For instance, salicylic acid features a single, sharp DOS peak located between −0.5 and 0 eV, originating from carbon p-states and, to a lesser extent, oxygen p-states. In contrast, acetylsalicylic acid exhibits a more complex structure with multiple peaks distributed between −1.0 and 0 eV for these same atomic and orbital contributions. For ibuprofen, the top of the valence band is overwhelmingly dominated by contributions from the 2p orbitals of carbon atoms, with a notably smaller contribution from oxygen 2p orbitals compared to the other crystals analyzed.

The conduction band minimum (CBM), corresponding to the lowest unoccupied molecular orbitals (LUMO), is consistently dominated by carbon 2p (pC) states across all four materials, suggesting that upon electronic excitation, the electron density would primarily localize on the carbon backbones. The conduction-state curves for salicylic acid and acetylsalicylic acid are qualitatively similar, disregarding the difference in their band gaps. A unique feature is observed in acetaminophen, which shows more significant contributions from hydrogen 1s orbitals near the bottom of the conduction band. These variations in the specific distributions of states can be attributed to the distinct molecular structure and functional groups in each crystal, which are expected to influence their optical properties and electronic conductivity.

The effective mass of charge carriers at a critical point (band extremum) ***k***_**0**_ of the *n*th band along a direction defined by the unit vector ***d̂*** is approximated using the parabolic dispersion relation

1 $$E_{n}\left(k_{0}+\epsilon \mathrm{d̂}\right)=E_{n}\left(k_{0}\right)+\frac{\hbar^{2}}{2m_{k_{0},\mathrm{d̂}}^{*}}{\left(\epsilon \mathrm{d̂}\right)}^{2}$$

where ϵ is sufficiently small to validate the parabolic approximation. Consequently, the effective mass *m*_***k***_**0**_, d̂_^*^ is inversely proportional to the curvature of the energy band at ***k***_**0**_. The computed effective masses for electrons and holes in the anti-inflammatory drug crystals are summarized in Table 3, reported in units of the free electron mass (*m*_0_), and evaluated along high-symmetry directions within the first Brillouin zone.

**Table 3:** Carrier Effective Masses (in Units of the Free Electron Mass) of Anti-inflammatory Crystals

effective mass	**Γ → A**	**Γ → B**	**Γ → Y**	**Γ → C**	**Γ → E**	**Γ → D**
Salicylic Acid						
*m*_e_	4.86	4.83	4.04	5.21	4.21	4.11
*m*_h_	2.23	1.92	14.32	9.04	2.13	1.78

	**Y → C**	**Y → Γ**	**A → E**	**A → Γ**	**E →Γ**	
Acetylsalicylic Acid						
*m*_e_	151.55	2.80	76.75	3.39	5.17	

	**B → A**	**B → D**	**B → Y**	**B → E**	**B → Γ**	
*m*_h_	9.93	5.34	2.56	10.19	12.76	

	**Γ → A**	**Γ → B**	**Γ → Y**	**Γ → C**	**Γ → E**	**Γ → D**
Acetaminophen						
*m*_e_	8.77	3.73	27.25	11.39	14.72	3.98
*m*_h_	5.49	2.49	5.18	3.85	51.5	24.52

	**B → D**	**B → Γ**	**Γ → Y**	**Γ → A**	**Γ → Z**	
Ibuprofen						
*m*_e_	3.02	42.23	14.25	17.96	2.81	
*m*_h_	3.13	5.03	5.02	10.19	4.00	

The results reveal pronounced anisotropy in the charge transport properties across all systems. In salicylic acid, the electron effective masses are moderately anisotropic, ranging from 4.04 *m*_0_ (Γ → *Y*) to 5.21 *m*_0_ (Γ→*C*). In contrast, the hole effective masses exhibit a stronger directional dependence. A notably heavy hole is found along the Γ → *Y* direction (*m*_*h*_ = 14.32 *m*_0_), whereas the Γ → *D* direction presents the lightest charge carrier in this crystal (*m*_*h*_ = 1.78 *m*_0_).

Acetylsalicylic acid shows the most extreme anisotropy, particularly for conduction band electrons. An exceptionally large electron effective mass of *m*_*e*_ = 151.55 *m*_0_ is calculated along the *Y → C* path, which suggests a nearly flat conduction band and, consequently, extremely low electron mobility in this direction. This contrasts sharply with the much lighter electron mass of 2.80 *m*_0_ along the *Y* → Γ direction. The hole effective masses are also anisotropic, though less extreme, with values between 2.56 *m*_0_ (*B* *→ Y*) and 12.76 *m*_0_ (*B* → Γ). In the case of acetaminophen, both carrier types show significant anisotropy. A very heavy hole is observed along the Γ → *E* direction with an effective mass of 51.5 *m*_0_. The electron masses are also highly variable, peaking at 27.25 *m*_0_ along the Γ → *C* direction. It is noteworthy that the Γ→*B* direction appears to be a preferential pathway for charge transport, exhibiting the lowest effective mass for both electrons (*m*_*e*_ = 3.73 *m*_0_) and holes (*m*_*h*_ = 2.49 *m*_0_).

Finally, ibuprofen is characterized by a significant anisotropy in its electron effective masses, which range from a relatively small 2.81 *m*_0_ (Γ → *Z*) to a large 42.23 *m*_0_ (*B* → Γ). Conversely, the holes in ibuprofen are comparatively lighter and more isotropic, with effective masses spanning a narrower range from 3.13 *m*_0_ (*B* *→* *D*) to 10.19 *m*_0_ (Γ → *A*). Overall, analysis of the effective masses indicates that charge transport in these anti-inflammatory crystals is highly dependent on the crystallographic direction.

It is essential to interpret the exceptionally large effective masses (e.g., *m*_*e*_ = 151.55 *m*_0_ for acetylsalicylic acid and *m*_*h*_ = 51.5 *m*_0_ for acetaminophen) reported in Table 3 with caution. These values are a direct mathematical result of the parabolic fitting method applied to extremely flat bands, as seen in Figures 6 and 7. Physically, they signify highly localized electronic states and near-zero group velocity along specific crystal directions. Effective masses exceeding 20 *m*_0_ generally indicate low-mobility, while values above 50 *m*_0_ render carrier contributions to macroscopic transport effectively negligible. This suggests that the conventional band-transport model is inappropriate for these carriers. Instead, charge transport in these directions would be dominated by a thermally activated hopping (polaron transport) mechanism between localized molecular orbitals, resulting in a very low carrier mobility. Our results thus correctly identify these pathways as electronically “insulating” from a band-transport perspective and highlight the critical role of crystal packing and intermolecular interactions in governing anisotropic electronic properties in organic pharmaceutical solids.

### 4.4 Optical Properties

Before discussing our results, it is important to note that the present DFT-based approach neglects excitonic interactions, which play a crucial role in the optical response of molecular crystals. Figure 8 shows the theoretical spectrum obtained in this work using DFT with Tkatchenko-Scheffler dispersion corrections (blue solid line). The experimental data, reported by Muthuselvi et al.,^115^ serves as a reliable benchmark for assessing the theoretical predictions. The computed spectrum reproduces the main experimental features, including multiple absorption peaks in the 4–6 eV range. However, the theoretical absorption onset appears near 3.5 eV, about 0.6 eV lower than the experimental onset at 4.1 eV. This systematic underestimation reflects the well-known limitations of semilocal DFT functionals, which describe electronic excitations in terms of independent Kohn-Sham particles and neglect electron–hole correlations. In molecular solids, where dielectric screening is weak, photoexcitation creates a correlated electron–hole pair that remains strongly bound by Coulomb attraction, forming an exciton. The corresponding binding energy (*E*_b_) lowers the absorption threshold, yielding an optical gap (*E*_g_^OPT^ = *E*_g_^QP^ – *E*_b_) smaller than the quasiparticle gap (*E*_g_^QP^) computed within DFT. This exciton binding can reach several hundred meV in low-dielectric materials, substantially modifying both the absorption onset and spectral intensity. Consequently, a quantitative description of optical transitions requires beyond-DFT methods that explicitly account for electron–hole interactions, such as the Bethe-Salpeter Equation (BSE) formalism,^116^ which consistently predicts smaller optical gaps and more accurate spectral profiles for molecular crystals.

**Figure 8:**
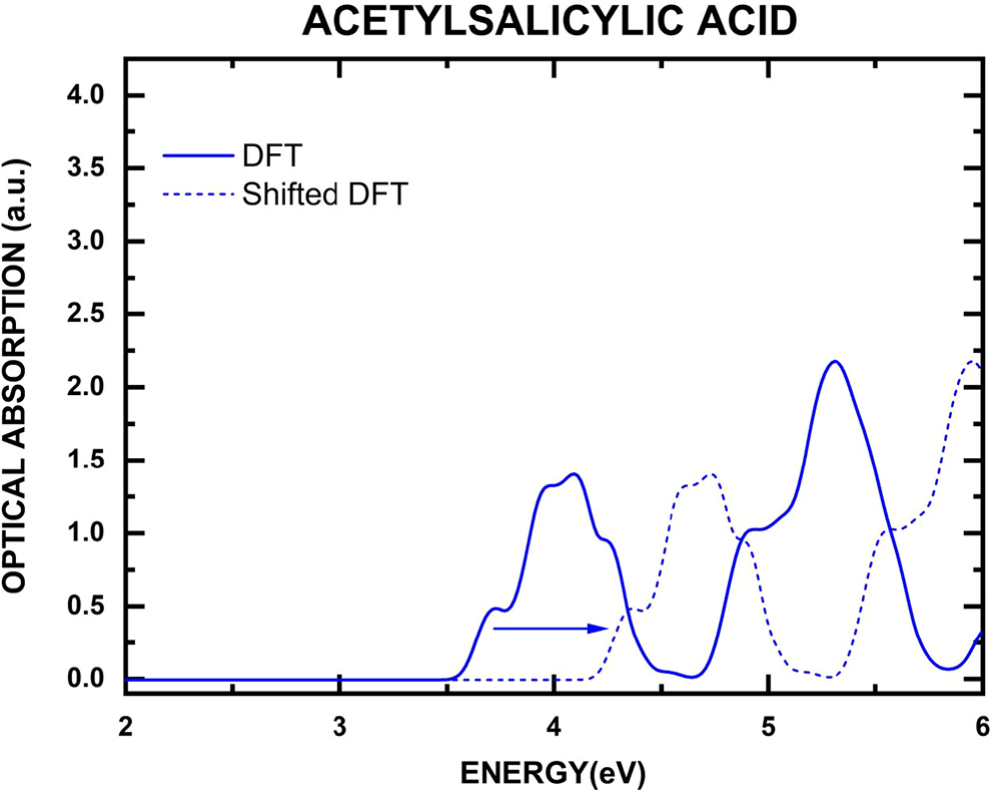
Optical absorption spectra of acetylsalicylic acid. The shifted DFT curve (dashed blue line) is obtained by translating the DFT spectrum to match the experimental onset of optical absorption (blue arrow) with the experimental curve reported in the work of Muthuselvi.^115^

In addition to excitonic effects, vibronic coupling and thermal lattice vibrations also have significant impacts on the optical properties of molecular solids. Our calculations are based on static 0 K geometries that neglect phonon contributions to electronic transitions. In real systems, however, the nuclei are not stationary: at finite temperatures, phonons dynamically modulate the electronic structure and interact with electronic excitations. This electron–phonon coupling leads to several experimentally observable consequences, including thermal broadening of absorption peaks, redistribution of spectral intensity, and the emergence of a subgap absorption tail known as the Urbach tail. The latter arises from phonon-assisted transitions that allow the system to absorb photons with energies slightly below the direct electronic gap. Vibronic effects can also shift peak positions and alter oscillator strengths, producing the smoother, more complex spectral features typically observed in experiments. Capturing these effects accurately would require either finite-temperature molecular dynamics coupled to electronic-structure sampling or perturbative treatments of electron–phonon interactions within the optical response function. Therefore, the sharper and slightly blue-shifted features in our calculated spectra, compared to the experimental data, are consistent with the neglect of both excitonic and vibronic effects in the present DFT-based framework.

To facilitate a more meaningful comparison of the spectral shapes, the theoretical spectrum was rigidly shifted by +0.6 eV, as indicated by the blue dashed line in Figure 6. After this energy correction, a much closer agreement between theoretical and experimental results is achieved. The shifted DFT spectrum accurately reproduces the position of the main experimental absorption peak observed near 4.8 eV and captures the secondary features at higher energies. Furthermore, the relative intensities of the absorption peaks after shifting are in reasonable agreement with the experimental data, although some discrepancies remain, likely attributable to the neglect of excitonic effects and many-body interactions in the DFT calculations.

The optical absorption spectra of the four monoclinic pharmaceutical compounds were computed for incident light polarized along three principal crystallographic directions (001, 010, and 100), as well as for a simulated polycrystalline sample (POLY), as shown in Figure 9. The results provide insights into the anisotropic optical properties of these materials and highlight key differences in their electronic structures.

**Figure 9:**
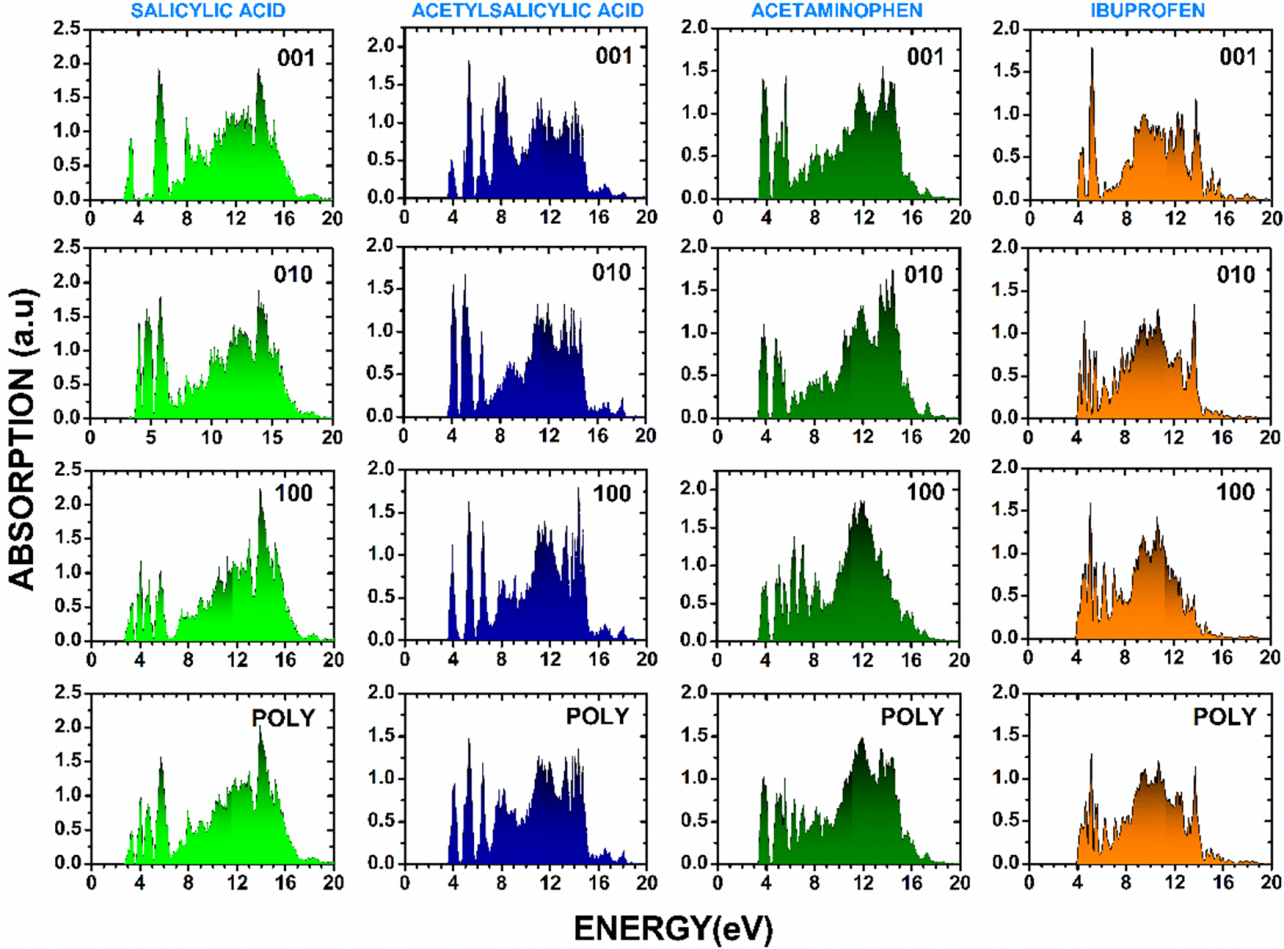
Optical absorption spectra of anti-inflammatory drug crystals considering incident light polarized along the 001, 010, and 100 crystal directions as well as incident light on a polycrystalline sample (POLY).

For salicylic acid, the absorption onset occurs at approximately 2.7 eV for the 100 polarization, with strong absorption peaks at 3.3, 4.1, 4.8, and 5.7 eV. Along the 010 direction, the onset shifts slightly to 2.8 eV, with a low-intensity peak at 3.4 eV and intense peaks at 4.1, 4.6, and 5.8 eV. The spectrum for the 001 polarization exhibits a prominent peak at 3.4 eV, followed by a broader absorption band extending from 5.2 to 6.5 eV, with a well-defined peak at 5.7 eV. The polycrystalline spectrum has an absorption onset at 2.8 eV and closely follows the spectral features observed in the 100 direction up to 7 eV. Compared to salicylic acid, the spectra of acetylsalicylic acid exhibit a higher degree of anisotropy, with an absorption onset at approximately 3.5 eV in the 001 case, with strong absorption peaks at 3.8, 5.3, and 6.4 eV. The 010 polarization shows a slightly higher onset at 3.6 eV, with intense peaks at 4.1, 5.1, and 6.5 eV. Similarly, the spectrum along 100 also has an onset at 3.6 eV, with dominant peaks at 3.9, 5.3, and 6.5 eV. The polycrystalline spectrum exhibits an absorption onset at 3.5 eV, averaging the key absorption features across all polarization cases.

Acetaminophen presents an absorption onset of approximately 3.3 eV for both the 001 and 010 polarized incident light. The 001 spectrum displays strong peaks at 3.7 and 5.5 eV, whereas the 010 spectrum exhibits intense peaks at 3.8 and 4.8 eV. The absorption onset along the (100) plane is slightly lower at 3.2 eV, with significant peaks at 4.0, 5.1, and 5.6 eV. The polycrystalline spectrum has an onset at 3.3 eV, closely resembling the averaged response of single-crystal orientations. Notably, the 001 and 010 spectra are more similar to each other than to the 100 spectra, particularly in the low-energy range.

Ibuprofen exhibits the highest absorption onset among the four compounds, with values of approximately 3.9 eV for the 001 and 100 polarizations and 4.0 eV for the 010 case. The 001 spectrum features peaks at 4.4 and 5.1 eV, while the 010 spectrum displays a series of closely spaced intense peaks at 4.3, 4.6, 5.1, 5.4, and 6.2 eV. The 100 configuration shows intense absorption at 4.7, 5.1, 5.6, and 6.3 eV. The polycrystalline spectrum has an absorption onset at 3.9 eV, with features resembling those of the 010 and 100 polarizations for energies up to 7 eV. Above 7 eV, the 001 and 010 spectra exhibit greater similarity to each other than to the 100 spectrum.

A comparative analysis of these absorption data highlights the significant anisotropy in the optical response of all four crystals. In general, the absorption spectra for light polarized along the 010 and 100 crystal directions show more pronounced fine structures, whereas the 001-oriented spectra tend to exhibit broader features. This anisotropic behavior suggests that electronic transitions in these materials are highly direction-dependent. The polycrystalline spectra, while smoothing out these directional dependencies, still preserve the essential absorption characteristics. The absorption features between 3 and 7 eV, which dominate all spectral curves, can be attributed to interband electronic transitions influenced by the molecular structures of the compounds. The observed anisotropy suggests potential applications in optoelectronic devices, where polarization-dependent absorption could be exploited. Further experimental validation of these computational results would be beneficial to confirm the predicted optical properties and explore potential technological applications.

The complex dielectric function, ε­(ω), describes the response of a material to an external electromagnetic field. It is defined as ε­(ω) = ε_1_(ω) + *i*ε_2_(ω), where ε_1_(ω) represents the real part and ε_2_(ω) represents the imaginary part. The real part, ε_1_(ω), provides information on the dispersive properties of the material, while the imaginary part, ε_2_(ω), is directly related to optical absorption through the material’s electronic transitions. The absorption coefficient previously calculated, α­(ω), is linked to ε­(ω) via the relation α(ω)=ωcIm⁡ε(ω) . High values of ε_2_(ω) indicate strong electronic transitions within the given energy range, leading to pronounced absorption features.

The real and imaginary parts of the dielectric function are interrelated through the Kramers–Kronig relations, which express the causality principle in the linear response theory. These relations state that the real part of the dielectric function can be obtained from the imaginary part and conversely, ε_2_(ω) can be derived from ε_1_(ω). The sign of ε_1_(ω) has important physical implications. A positive value indicates that the material supports propagating electromagnetic waves and exhibits normal dispersion. Negative values correspond to frequency ranges where the material behaves like a metal, preventing wave propagation and leading to reflection or absorption. These negative values often occur near strong electronic transitions or plasmonic resonances, signifying a region where the material exhibits metallic-like optical properties. In molecular crystals, such a behavior is typically associated with charge-transfer excitations and strong electron–phonon interactions.

Figure 10 depicts ε_1_(ω) (black curves) and ε_2_(ω) (orange curves) for each anti-inflammatory crystal under study, considering incident polarized light parallel to the (001), (010), and (100) crystal directions as well as light incident on a polycrystalline sample (POLY), following the same approach used in the optical absorption calculations. Since the imaginary part, ε_2_(ω), is more directly related to the optical absorption previously discussed, we focus here on providing a detailed description of the real part, ε_1_(ω). For salicylic acid, the real part of the dielectric function exhibits strong anisotropy across different polarization directions. In the 001 polarization, ε_1_(0) = 2.6, with maxima at 2.9 eV (5.6), 5.3 eV (7.4), and 7.8 eV (2.9), while negative values appear between 3.3 and 3.6 eV. In contrast, the 010 polarization has a higher static dielectric constant, ε_1_(0) = 2.9, and presents strong peaks at 3.8 eV (10) and 4.4 eV (6.6), followed by negative regions between 4.0 and 6.5 eV. The 100 polarization plane is characterized by a slightly lower ε_1_(0) and negative values between 4.0 and 6.0 eV, while the polycrystalline sample exhibits smoother variations, with the most prominent maximum at 3.7 eV. One can infer that the dielectric response is strongest along the 010 polarization and weakest along the 100 direction. This observation correlates directly with the orientation of the primary chromophore of this molecule, the phenol group. The crystallographic data reveal that the phenol group plane is most aligned with the 010 polarization vector. Consequently, an electric field oscillating along this axis is maximally effective at inducing the polarizable π → π* electronic transitions within the aromatic system, leading to the intense peak observed in ε_1_(ω). The O1–H6···O3 and O2–H5···O3 hydrogen bonds that form the dimer motif of the crystal, on the other hand, are most aligned with the 100 direction. Therefore, the transitions associated with the aromatic ring are clearly dominant in this energy range.

**Figure 10:**
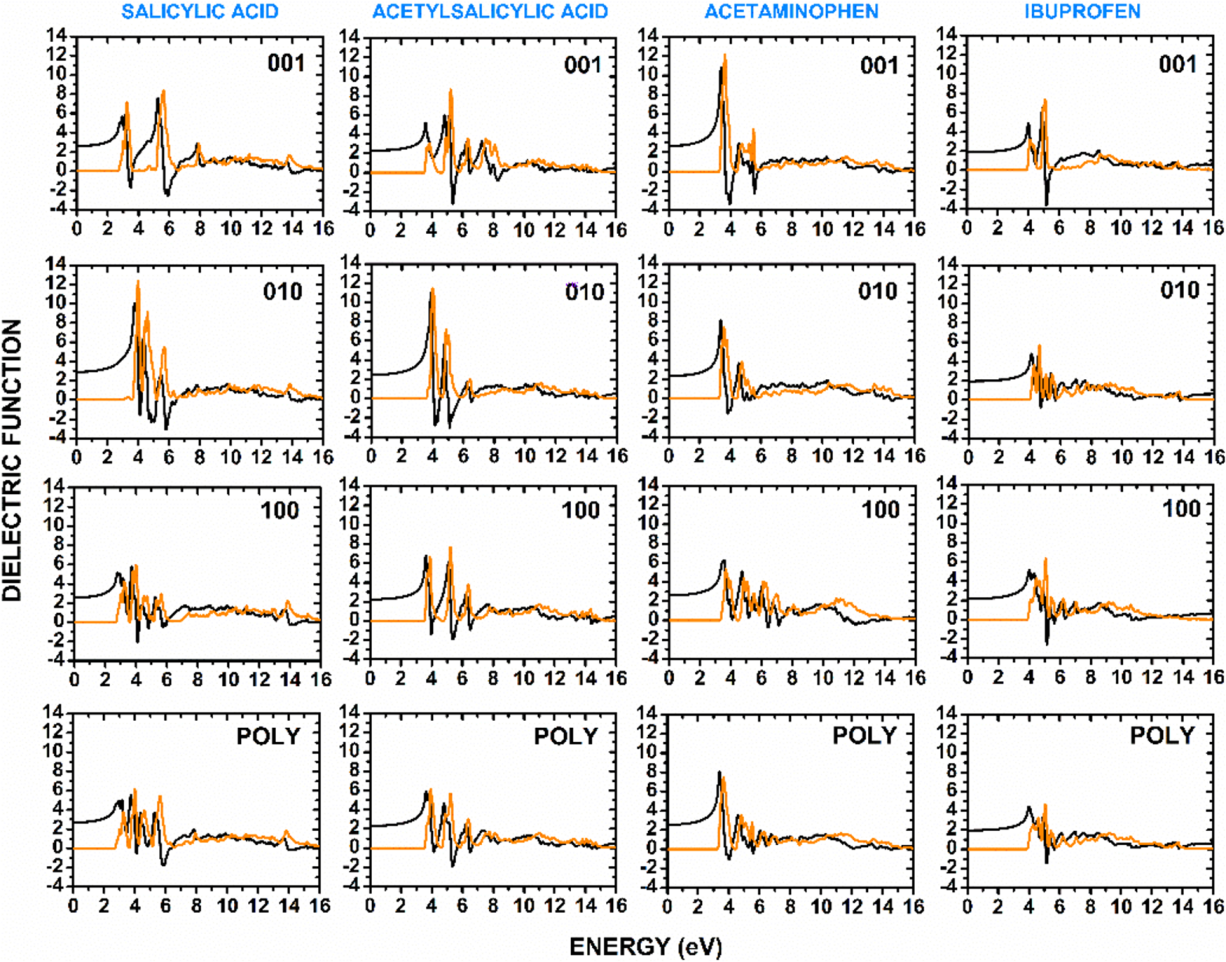
Complex dielectric function ε­(ω) = ε_1_(ω) + *i*ε_2_(ω) of anti-inflammatory drug crystals considering incident light polarized along the 001, 010, and 100 crystal directions as well as incident light on a polycrystalline sample (POLY). The black curves correspond to the real part ε_1_(ω), while the orange curves depict ε_2_(ω). The plots highlight the strong dielectric anisotropy with different responses for light polarized along the three principal crystal directions.

Acetylsalicylic acid shows a similar but slightly weaker anisotropic behavior. For the 001 polarization, ε_1_(0) = 2.3, with peaks at 3.6 eV (5.2), 4.9 eV (5.9), and 7.3 eV (3.3). Negative values occur between 5.3 and 8.5 eV. The 010 polarization presents a significant peak at 3.9 eV (10.9), while negative regions extend between 4.1 and 5.7 eV. The 100 polarization follows a comparable pattern but with slightly lower magnitudes. The polycrystalline sample smooths out the differences between polarizations but still retains a peak at 3.6 eV (5.9) and a negative region between 5.2 and 5.7 eV. These observations correlate directly with the orientation of the benzene ring. The crystallographic data reveal that the phenol group plane is most aligned with the 010 polarization vector. Consequently, an electric field oscillating along this axis is maximally effective at inducing the polarizable π → π* electronic transitions within the aromatic system, leading to the intense peaks observed in ε_1_(ω). In addition, the O1–H1···O8 hydrogen bonds that form the dimer motif of the crystal are also most aligned with the 010 direction, reinforcing the transitions associated with the benzene ring.

In the case of acetaminophen, anisotropy is again evident. The 001 polarization exhibits a strong peak at 3.4 eV (10.9) and a significant negative region between 3.7 and 5.8 eV. The 010 polarization has a lower static value of ε_1_(0) = 2.3 and presents peaks at 3.4 eV (8.1) and 4.6 eV (3.7), while the negative region is limited to 3.8 and 4.1 eV. The 100 polarization, in contrast, is characterized by a broad distribution of peaks and minima, with negative values occurring near the local minima. The polycrystalline sample follows a trend like the averaged behavior of the single-crystal orientations, maintaining negative values around 3.8–4.2 eV. Acetaminophen presents a unique case where the dielectric anisotropy is governed by intramolecular charge transfer. The highest intensity peak in ε_1_(ω) is observed along the 001 direction, even though the phenol plane and the hydrogen bond O1–H1···O2 are mostly aligned with the 010 and 100 axes, respectively. The key to this behavior lies in its HOMO–LUMO transition, which involves a significant charge transfer from the electron-donating hydroxyl group to the electron-accepting amide group. The transition dipole moment for this specific excitation is oriented along this intramolecular phenol-to-amide vector. Within the crystal, this vector is preferentially aligned with the 001 crystallographic axis. Therefore, light polarized along 001 most effectively drives this charge-transfer transition, leading to the dominant peak in the dielectric function. Some contribution from the N1–H6···O1 hydrogen bond also helps to reinforce this behavior, as this bond is most aligned along 001.

For ibuprofen, the dielectric function exhibits the lowest static dielectric constant among the studied materials. The 001 polarization has ε_1_(0) = 1.9 and a peak at 4.9 eV (6.4), with a negative region between 5.1 and 5.5 eV. The 010 and 100 polarizations follow similar trends, with their most notable peaks around 4.0 and 4.9 eV. The polycrystalline response is relatively smooth, but negative values persist between 5.1 and 5.3 eV. This anisotropy is a clear reflection of its molecular packing. In the ibuprofen crystal, both the phenyl group plane and the hydrogen bonds O1–H1···O2 of the carboxylic acid dimer are most aligned with the 100 direction. An electric field oriented along this axis can thus effectively polarize all the primary chromophore components of the molecule. The weaker responses in the other two directions correspond to their less favorable alignment with these molecular features. While the response of ibuprofen directly mirrors its structural orientation, the overall magnitude of ε_1_(ω) remains the lowest of the four compounds, a characteristic attributed to the ″dilution effect″ of its large, nonpolar isobutyl group, which increases the unit cell volume and lowers the density of polarizable moieties.

Comparing the four materials, it is evident that acetaminophen and acetylsalicylic acid exhibit the strongest dielectric responses, particularly along the 010 direction, where peaks reach values as high as ε_1_(ω) = 10.9. Negative values are present in all materials but are more prominent in salicylic and acetylsalicylic acid. The polycrystalline samples tend to average out the sharp variations seen in the single-crystal orientations, yielding a more uniform response across the energy spectrum.

## 5 Conclusions

In this work, we have successfully employed density functional theory (DFT) with dispersion corrections to elucidate the structural, electronic, and optical properties of the monoclinic crystalline forms of salicylic acid, acetylsalicylic acid, acetaminophen, and ibuprofen, four cornerstone pharmaceuticals with significant clinical relevance. Our geometry optimizations, conducted using the GGA-PBE functional augmented by the Tkatchenko–Scheffler (TS) dispersion correction, yielded lattice parameters and unit cell volumes in close agreement with experimental crystallographic data, with deviations typically within 1–2%. These results underscore the efficacy of the TS correction in capturing van der Waals interactions critical to molecular crystals, though minor discrepancies, such as the underestimation of the unit cell volume of acetaminophen by 2.2% and ibuprofen by 1.88%, highlight the limitations of semilocal functionals in fully resolving anisotropic intermolecular forces. Future studies could benefit from hybrid functionals or advanced dispersion methods like many-body dispersion (MBD) to further refine these predictions, albeit at a higher computational cost.

TD-DFT simulations accurately reproduced molecular UV–vis absorption peaks, e.g., salicylic acid and acetylsalicylic acid HOMO → LUMO transitions at 278 nm (4.46 eV), aligning well with the experimental literature. The electronic band structure calculations using the meta-GGA HLE17 functional for the solid-state systems revealed indirect band gaps of 2.99 eV for salicylic acid, 3.76 eV for acetylsalicylic acid, 3.56 eV for acetaminophen, and 4.02 eV for ibuprofen, consistent with their insulating nature as molecular crystals. Notably, the presence of near-direct gaps, particularly in salicylic acid, acetaminophen, and ibuprofen, suggests that optical transitions at the Γ point may contribute significantly to their optoelectronic behavior. These electronic properties are intricately linked to the molecular conformations and hydrogen-bonding motifs within the crystals, as evidenced by the flatness of bands in acetylsalicylic acid (indicating localized states) versus the greater dispersion in ibuprofen (suggesting enhanced charge mobility).

The comparison between the experimental and theoretical optical absorption spectra of acetylsalicylic acid confirms that DFT calculations within the GGA-PBE+TS framework can reliably capture the main features of the optical response despite the systematic underestimation of the absorption onset. These results emphasize the importance of applying appropriate energy corrections and point to the potential benefits of employing many-body approaches for more accurate optical property predictions in molecular crystals. Optical property analyses further revealed pronounced anisotropy in the absorption spectra and complex dielectric functions along the (100), (010), and (001) crystallographic directions, reflecting the directional dependence of intermolecular forces due to hydrogen bonding, interactions involving the π states of the aromatic ring, and van der Waals forces. For instance, the salicylic acid crystal exhibits stronger absorption below 5 eV along the plane (010), which is aligned with the intramolecular hydrogen bond directions within the respective unit cells, while for the acetylsalicylic acid crystal, a similar behavior seems to be modulated by C–H···π and C=O···π interactions. These anisotropic optical characteristics have implications for the photochemical stability of these compounds under varying environmental conditions, which is a key consideration in pharmaceutical storage and delivery.

Collectively, our findings connect the molecular-level understanding of these drugs with their macroscopic solid-state behavior. This provides a foundation for optimizing their crystalline forms and formulation. Specifically, the calculated properties suggest several application-relevant insights. The wide, indirect band gaps confirm their nature as robust insulators, making them suitable as potential dielectric layers in organic electronics, although their low charge mobility would be a limitation. More relevant to pharmaceutics, their strong optical absorption, particularly in the UV-C and UV-B regions (Figure 9), provides a quantitative basis for their known photochemical instability. This underscores the necessity for UV-blocking packaging for these solid-state formulations.

Furthermore, the pronounced optical anisotropy (Figures 9 and 10) implies that the orientation of microcrystals in a formulation (e.g., in a cream or compressed tablet) could significantly affect light-induced degradation rates. For example, the high absorption of salicylic acid along the 010 direction suggests that crystallites with this orientation would be most vulnerable to degradation. While near-direct gaps might hint at the optoelectronic potential, such as their use in photodetectors, our effective mass analysis reveals clear limitations. The presence of extremely heavy carriers in acetylsalicylic acid and acetaminophen implies very low charge mobility, making them poor candidates for applications requiring efficient charge transport. This highlights the power of DFT-based approaches not only for identifying potential but also for anticipating the limitations of molecular materials in solid-state applications. Future work could extend these simulations to polymorphs, cocrystals, or amorphous phases, incorporating temperature effects or solvent interactions to further align computational predictions with real-world pharmaceutical applications.

## Supplementary Material



## Data Availability

Data will be made available on request.

## Notes

Optimized and experimental atoms coordinates for all crystalline systems studied in this work (PDF)
