# Supplementary material for: Structural, Electronic, and Optical Properties of Monoclinic Pharmaceutical Crystals: A DFT Study of Salicylic Acid, Acetylsalicylic Acid, Acetaminophen, and Ibuprofen
Source: ACS Omega. 2026 Jan 26;11(5):7074–92. doi: 10.1021/acsomega.5c06006 (PMC12903142; doi:10.1021/acsomega.5c06006)
Supplement: Supplementary file 1 [file ao5c06006_si_001.pdf]

Structural, Electronic, and Optical Properties of Monoclinic Pharmaceutical Crystals: A DFT Study of Salicylic Acid, Acetylsalicylic Acid, Acetaminophen, and Ibuprofen

Liciane L. Ferreira[a], Mariana S. Alves[a], Micael E. P. Oliveira[a], Valder N. Freire[a], Bruno P. Silva[b*], José B. Silva[c], Ewerton W. S. Caetano[d*]

[a] Departamento de Física, Universidade Federal do Ceará, Caixa Postal 6030, 60440-900, Fortaleza-CE Brazil

[b] Computational and Quantum Chemistry Group, Instituto Federal de Educação, Ciência e Tecnologia do Ceará, Campus Camocim, 62400-000, Camocim-CE, Brazil

[c] Departamento de Química Biológica, Universidade Regional do Cariri, 63105-000, Crato-CE Brazil

[d] Departamento de Física e Matemática, Instituto Federal de Educação, Ciência e Tecnologia do Ceará, Campus Fortaleza, 60040-531, Fortaleza-CE, Brazil

[*]Bruno Poti e Silva email: bruno.poti@ifce.edu.br

[*]Ewerton Wagner Santos Caetano email: ewcaetano@gmail.com

SUPPORTING INFORMATION

Table S1: Atom coordinates relative to the unit cell lattice parameters for Salicylic Acid.

Salicylic acid						
	Experimental data			Geometry optimization		
Element	x	y	z	x	y	z
H1	0.573110	0.316970	-0.057130	0.570859	0.316593	-0.059044
H2	0.040640	0.228800	0.296170	0.041173	0.228498	0.297680
H3	0.215410	0.457370	0.001560	0.218645	0.459430	0.005316
H4	-0.043120	0.415340	0.184300	-0.050918	0.414636	0.184324
H5	0.540710	0.021760	0.218980	0.547063	0.022770	0.210269
H6	0.983720	0.075980	-0.070490	0.996332	0.074415	-0.068198
H7	-0.573110	0.816970	0.557130	-0.570859	0.816593	0.559044
H8	-0.040640	0.728800	0.203830	-0.041173	0.728498	0.202320
H9	-0.215410	0.957370	0.498440	-0.218645	0.959430	0.494684
H10	0.043120	0.915340	0.315700	0.050918	0.914636	0.315676
H11	-0.540710	0.521760	0.281020	-0.547063	0.522770	0.289731
H12	-0.983720	0.575980	0.570490	-0.996332	0.574415	0.568198
H13	-0.573110	-0.316970	0.057130	-0.570859	-0.316593	0.059044
H14	-0.040640	-0.228800	-0.296170	-0.041173	-0.228498	-0.297680
H15	-0.215410	-0.457370	-0.001560	-0.218645	-0.459430	-0.005316
H16	0.043120	-0.415340	-0.184300	0.050918	-0.414636	-0.184324
H17	-0.540710	-0.021760	-0.218980	-0.547063	-0.022770	-0.210269
H18	-0.983720	-0.075980	0.070490	-0.996332	-0.074415	0.068198
H19	0.573110	0.183030	0.442870	0.570859	0.183407	0.440956
H20	0.040640	0.271200	0.796170	0.041173	0.271502	0.797680
H21	0.215410	0.042630	0.501560	0.218645	0.040570	0.505316
H22	-0.043120	0.084660	0.684300	-0.050918	0.085364	0.684324
H23	0.540710	0.478240	0.718980	0.547063	0.477230	0.710269
H24	0.983720	0.424020	0.429510	0.996332	0.425585	0.431802
C1	0.505160	0.192840	0.082380	0.507720	0.192244	0.081779
C2	0.158730	0.249240	0.220210	0.155920	0.248786	0.219560
C3	0.453700	0.299280	0.019080	0.453911	0.298192	0.018812
C4	0.356090	0.167770	0.184100	0.356587	0.167615	0.183889
C5	0.258860	0.379610	0.055410	0.255964	0.377912	0.054985
C6	0.111040	0.353990	0.156480	0.106482	0.352609	0.155633
C7	0.713980	0.109080	0.043970	0.718050	0.108884	0.044178
C8	-0.505160	0.692840	0.417620	-0.507720	0.692244	0.418221
C9	-0.158730	0.749240	0.279790	-0.155920	0.748786	0.280440
C10	-0.453700	0.799280	0.480920	-0.453911	0.798192	0.481188
C11	-0.356090	0.667770	0.315900	-0.356587	0.667615	0.316111
C12	-0.258860	0.879610	0.444590	-0.255964	0.877912	0.445015
C13	-0.111040	0.853990	0.343520	-0.106482	0.852609	0.344367
C14	-0.713980	0.609080	0.456030	-0.718050	0.608884	0.455822
C15	-0.505160	-0.192840	-0.082380	-0.507720	-0.192244	-0.081779
C16	-0.158730	-0.249240	-0.220210	-0.155920	-0.248786	-0.219560
C17	-0.453700	-0.299280	-0.019080	-0.453911	-0.298192	-0.018812
C18	-0.356090	-0.167770	-0.184100	-0.356587	-0.167615	-0.183889
C19	-0.258860	-0.379610	-0.055410	-0.255964	-0.377912	-0.054985
C20	-0.111040	-0.353990	-0.156480	-0.106482	-0.352609	-0.155633
C21	-0.713980	-0.109080	-0.043970	-0.718050	-0.108884	-0.044178
C22	0.505160	0.307160	0.582380	0.507720	0.307756	0.581779
C23	0.158730	0.250760	0.720210	0.155920	0.251214	0.719560

C24	0.453700	0.200720	0.519080	0.453911	0.201808	0.518812
C25	0.356090	0.332230	0.684100	0.356587	0.332385	0.683889
C26	0.258860	0.120390	0.555410	0.255964	0.122088	0.554985
C27	0.111040	0.146010	0.656480	0.106482	0.147391	0.655633
C28	0.713980	0.390920	0.543970	0.718050	0.391116	0.544178
O1	0.839990	0.139280	-0.052150	0.848290	0.136649	-0.051574
O2	0.392750	0.067560	0.249990	0.398428	0.068128	0.248861
O3	0.767620	0.014830	0.098970	0.770428	0.014418	0.100895
O4	-0.839990	0.639280	0.552150	-0.848290	0.636649	0.551574
O5	-0.392750	0.567560	0.250010	-0.398428	0.568128	0.251139
O6	-0.767620	0.514830	0.401030	-0.770428	0.514418	0.399105
O7	-0.839990	-0.139280	0.052150	-0.848290	-0.136649	0.051574
O8	-0.392750	-0.067560	-0.249990	-0.398428	-0.068128	-0.248861
O9	-0.767620	-0.014830	-0.098970	-0.770428	-0.014418	-0.100895
O10	0.839990	0.360720	0.447850	0.848290	0.363351	0.448426
O11	0.392750	0.432440	0.749990	0.398428	0.431872	0.748861
O12	0.767620	0.485170	0.598970	0.770428	0.485582	0.600895

Table S2: Atom coordinates relative to the unit cell lattice parameters for Acetylsalicylic Acid.

Acetylsalicylic acid

	Experimental data			Geometry optimization		
Element	x	y	z	x	y	z
H1	0.372100	0.239900	-0.008100	0.370809	0.237393	-0.006567
H2	0.302500	0.041400	0.159700	0.303823	0.034413	0.159255
H3	0.138500	0.176500	0.270700	0.139728	0.170788	0.266790
H4	0.042600	0.505000	0.207200	0.043813	0.502428	0.204660
H5	0.462100	0.960900	-0.149800	0.454650	0.975783	-0.145116
H6	0.319400	0.898000	-0.223300	0.319167	0.879092	-0.227167
H7	0.439100	0.734100	-0.231300	0.452004	0.733819	-0.219676
H8	0.072000	0.984300	-0.067300	0.068328	0.991189	-0.065218
H9	-0.372100	0.739900	0.508100	-0.370809	0.737393	0.506567
H10	-0.302500	0.541400	0.340300	-0.303823	0.534413	0.340745
H11	-0.138500	0.676500	0.229300	-0.139728	0.670788	0.233210
H12	-0.042600	1.005000	0.292800	-0.043813	1.002428	0.295340
H13	-0.462100	1.460900	0.649800	-0.454650	1.475783	0.645116
H14	-0.319400	1.398000	0.723300	-0.319167	1.379092	0.727167
H15	-0.439100	1.234100	0.731300	-0.452004	1.233819	0.719676
H16	-0.072000	1.484300	0.567300	-0.068328	1.491189	0.565218
H17	-0.372100	-0.239900	0.008100	-0.370809	-0.237393	0.006567
H18	-0.302500	-0.041400	-0.159700	-0.303823	-0.034413	-0.159255
H19	-0.138500	-0.176500	-0.270700	-0.139728	-0.170788	-0.266790
H20	-0.042600	-0.505000	-0.207200	-0.043813	-0.502428	-0.204660
H21	-0.462100	-0.960900	0.149800	-0.454650	-0.975783	0.145116
H22	-0.319400	-0.898000	0.223300	-0.319167	-0.879092	0.227167
H23	-0.439100	-0.734100	0.231300	-0.452004	-0.733819	0.219676
H24	-0.072000	-0.984300	0.067300	-0.068328	-0.991189	0.065218
H25	0.372100	0.260100	0.491900	0.370809	0.262607	0.493433
H26	0.302500	0.458600	0.659700	0.303823	0.465587	0.659255
H27	0.138500	0.323500	0.770700	0.139728	0.329212	0.766790
H28	0.042600	-0.005000	0.707200	0.043813	-0.002428	0.704660
H29	0.462100	-0.460900	0.350200	0.454650	-0.475783	0.354884
H30	0.319400	-0.398000	0.276700	0.319167	-0.379092	0.272833
H31	0.439100	-0.234100	0.268700	0.452004	-0.233819	0.280324
H32	0.072000	-0.484300	0.432700	0.068328	-0.491189	0.434782
C1	0.153660	0.559600	0.066760	0.153517	0.560594	0.067389
C2	0.247650	0.481500	0.007430	0.247110	0.481806	0.009278
C3	0.300300	0.295900	0.040400	0.299798	0.293551	0.041589
C4	0.261200	0.185500	0.134800	0.261895	0.181423	0.134557
C5	0.168300	0.260700	0.195300	0.170079	0.256762	0.194226
C6	0.115290	0.445100	0.160830	0.116511	0.443292	0.160119
C7	0.090100	0.755400	0.037660	0.088940	0.756750	0.038054
C8	0.367160	0.737300	-0.063240	0.371267	0.735592	-0.059558
C9	0.399100	0.841100	-0.173600	0.400990	0.838965	-0.169615
C10	-0.153660	1.059600	0.433240	-0.153517	1.060594	0.432611
C11	-0.247650	0.981500	0.492570	-0.247110	0.981806	0.490722
C12	-0.300300	0.795900	0.459600	-0.299798	0.793551	0.458411
C13	-0.261200	0.685500	0.365200	-0.261895	0.681423	0.365443
C14	-0.168300	0.760700	0.304700	-0.170079	0.756762	0.305774
C15	-0.115290	0.945100	0.339170	-0.116511	0.943292	0.339881
C16	-0.090100	1.255400	0.462340	-0.088940	1.256750	0.461946
C17	-0.367160	1.237300	0.563240	-0.371267	1.235592	0.559558
C18	-0.399100	1.341100	0.673600	-0.400990	1.338965	0.669615
C19	-0.153660	-0.559600	-0.066760	-0.153517	-0.560594	-0.067389

C20	-0.247650	-0.481500	-0.007430	-0.247110	-0.481806	-0.009278
C21	-0.300300	-0.295900	-0.040400	-0.299798	-0.293551	-0.041589
C22	-0.261200	-0.185500	-0.134800	-0.261895	-0.181423	-0.134557
C23	-0.168300	-0.260700	-0.195300	-0.170079	-0.256762	-0.194226
C24	-0.115290	-0.445100	-0.160830	-0.116511	-0.443292	-0.160119
C25	-0.090100	-0.755400	-0.037660	-0.088940	-0.756750	-0.038054
C26	-0.367160	-0.737300	0.063240	-0.371267	-0.735592	0.059558
C27	-0.399100	-0.841100	0.173600	-0.400990	-0.838965	0.169615
C28	0.153660	-0.059600	0.566760	0.153517	-0.060594	0.567389
C29	0.247650	0.018500	0.507430	0.247110	0.018194	0.509278
C30	0.300300	0.204100	0.540400	0.299798	0.206449	0.541589
C31	0.261200	0.314500	0.634800	0.261895	0.318577	0.634557
C32	0.168300	0.239300	0.695300	0.170079	0.243238	0.694226
C33	0.115290	0.054900	0.660830	0.116511	0.056708	0.660119
C34	0.090100	-0.255400	0.537660	0.088940	-0.256750	0.538054
C35	0.367160	-0.237300	0.436760	0.371267	-0.235592	0.440442
C36	0.399100	-0.341100	0.326400	0.400990	-0.338965	0.330385
O1	0.123200	0.858100	-0.053300	0.120861	0.862097	-0.051879
O2	0.010100	0.813300	0.095600	0.008855	0.814324	0.096375
O3	0.288800	0.581800	-0.089400	0.287311	0.583543	-0.086451
O4	0.404400	0.781000	0.037700	0.413645	0.772991	0.041390
O5	-0.123200	1.358100	0.553300	-0.120861	1.362097	0.551879
O6	-0.010100	1.313300	0.404400	-0.008855	1.314324	0.403625
O7	-0.288800	1.081800	0.589400	-0.287311	1.083543	0.586451
O8	-0.404400	1.281000	0.462300	-0.413645	1.272991	0.458610
O9	-0.123200	-0.858100	0.053300	-0.120861	-0.862097	0.051879
O10	-0.010100	-0.813300	-0.095600	-0.008855	-0.814324	-0.096375
O11	-0.288800	-0.581800	0.089400	-0.287311	-0.583543	0.086451
O12	-0.404400	-0.781000	-0.037700	-0.413645	-0.772991	-0.041390
O13	0.123200	-0.358100	0.446700	0.120861	-0.362097	0.448121
O14	0.010100	-0.313300	0.595600	0.008855	-0.314324	0.596375
O15	0.288800	-0.081800	0.410600	0.287311	-0.083543	0.413549
O16	0.404400	-0.281000	0.537700	0.413645	-0.272991	0.541390

Table S3: Atom coordinates relative to the unit cell lattice parameters for Acetominophen.

Acetominophen						
	Experimental data			Geometry optimization		
Element	x	y	z	x	y	z
H1	0.051500	0.957300	0.241400	0.032235	0.970302	0.238456
H2	-0.157865	0.902158	0.071669	-0.168998	0.898082	0.065929
H3	-0.198242	0.733778	-0.100332	-0.207522	0.726865	-0.099896
H4	0.378585	0.567606	-0.000300	0.374436	0.578166	-0.001279

H5	0.418477	0.736194	0.171672	0.412731	0.746736	0.165092
H6	-0.034300	0.429200	-0.343600	-0.065468	0.418283	-0.350777
H7	0.100100	0.303400	-0.305500	0.111081	0.280867	-0.308577
H8	0.161100	0.420700	-0.385800	0.163693	0.423769	-0.399686
H9	1.095809	0.472457	0.197029	1.095810	0.459310	0.197553
H10	0.448500	1.457300	0.258600	0.467765	1.470302	0.261544
H11	0.657865	1.402158	0.428331	0.668998	1.398082	0.434071
H12	0.698242	1.233778	0.600332	0.707522	1.226865	0.599896
H13	0.121415	1.067606	0.500300	0.125564	1.078166	0.501279
H14	0.081523	1.236194	0.328328	0.087269	1.246736	0.334908
H15	0.534300	0.929200	0.843600	0.565468	0.918283	0.850777
H16	0.399900	0.803400	0.805500	0.388919	0.780867	0.808577
H17	0.338900	0.920700	0.885800	0.336307	0.923769	0.899686
H18	-0.595809	0.972457	0.302971	-0.595810	0.959310	0.302447
H19	-0.051500	-0.957300	-0.241400	-0.032235	-0.970302	-0.238456
H20	0.157865	-0.902158	-0.071669	0.168998	-0.898082	-0.065929
H21	0.198242	-0.733778	0.100332	0.207522	-0.726865	0.099896
H22	-0.378585	-0.567606	0.000300	-0.374436	-0.578166	0.001279
H23	-0.418477	-0.736194	-0.171672	-0.412731	-0.746736	-0.165092
H24	0.034300	-0.429200	0.343600	0.065468	-0.418283	0.350777
H25	-0.100100	-0.303400	0.305500	-0.111081	-0.280867	0.308577
H26	-0.161100	-0.420700	0.385800	-0.163693	-0.423769	0.399686
H27	-1.095809	-0.472457	-0.197029	-1.095810	-0.459310	-0.197553
H28	0.551500	-0.457300	0.741400	0.532235	-0.470302	0.738456
H29	0.342135	-0.402158	0.571669	0.331002	-0.398082	0.565929
H30	0.301758	-0.233778	0.399668	0.292478	-0.226865	0.400104
H31	0.878585	-0.067606	0.499700	0.874436	-0.078166	0.498721
H32	0.918477	-0.236194	0.671672	0.912731	-0.246736	0.665092
H33	0.465700	0.070800	0.156400	0.434532	0.081717	0.149223
H34	0.600100	0.196600	0.194500	0.611081	0.219133	0.191423
H35	0.661100	0.079300	0.114200	0.663693	0.076231	0.100314
H36	1.595809	0.027543	0.697029	1.595810	0.040690	0.697553
C1	0.134600	0.829400	0.129680	0.125757	0.836239	0.126165
C2	-0.036500	0.827700	0.054960	-0.049771	0.828160	0.051576
C3	-0.058500	0.735500	-0.039390	-0.070930	0.732679	-0.042185
C4	0.088800	0.643800	-0.061370	0.081836	0.643348	-0.063728
C5	0.258100	0.643700	0.014970	0.255796	0.648493	0.012435
C6	0.280000	0.736200	0.109540	0.277222	0.744694	0.106183
C7	0.179200	0.488200	-0.219420	0.175156	0.485006	-0.221654
C8	0.093400	0.402300	-0.323400	0.088628	0.398062	-0.326389
C9	0.365400	1.329400	0.370320	0.374243	1.336239	0.373835
C10	0.536500	1.327700	0.445040	0.549771	1.328160	0.448424
C11	0.558500	1.235500	0.539390	0.570930	1.232679	0.542185
C12	0.411200	1.143800	0.561370	0.418164	1.143348	0.563728
C13	0.241900	1.143700	0.485030	0.244204	1.148493	0.487565
C14	0.220000	1.236200	0.390460	0.222778	1.244694	0.393817
C15	0.320800	0.988200	0.719420	0.324844	0.985006	0.721654
C16	0.406600	0.902300	0.823400	0.411372	0.898062	0.826389
C17	-0.134600	-0.829400	-0.129680	-0.125757	-0.836239	-0.126165
C18	0.036500	-0.827700	-0.054960	0.049771	-0.828160	-0.051576
C19	0.058500	-0.735500	0.039390	0.070930	-0.732679	0.042185
C20	-0.088800	-0.643800	0.061370	-0.081836	-0.643348	0.063728
C21	-0.258100	-0.643700	-0.014970	-0.255796	-0.648493	-0.012435

C22	-0.280000	-0.736200	-0.109540	-0.277222	-0.744694	-0.106183
C23	-0.179200	-0.488200	0.219420	-0.175156	-0.485006	0.221654
C24	-0.093400	-0.402300	0.323400	-0.088628	-0.398062	0.326389
C25	0.634600	-0.329400	0.629680	0.625757	-0.336239	0.626165
C26	0.463500	-0.327700	0.554960	0.450229	-0.328160	0.551576
C27	0.441500	-0.235500	0.460610	0.429070	-0.232679	0.457815
C28	0.588800	-0.143800	0.438630	0.581836	-0.143348	0.436272
C29	0.758100	-0.143700	0.514970	0.755796	-0.148493	0.512435
C30	0.780000	-0.236200	0.609540	0.777222	-0.244694	0.606183
C31	0.679200	0.011800	0.280580	0.675156	0.014994	0.278346
C32	0.593400	0.097700	0.176600	0.588628	0.101938	0.173611
N1	0.052800	0.552900	-0.159890	0.046660	0.550349	-0.162037
N2	0.447200	1.052900	0.659890	0.453340	1.050349	0.662037
N3	-0.052800	-0.552900	0.159890	-0.046660	-0.550349	0.162037
N4	0.552800	-0.052900	0.340110	0.546660	-0.050349	0.337963
O1	0.164900	0.921110	0.223120	0.155803	0.931563	0.217232
O2	0.353200	0.496350	-0.191410	0.353080	0.494937	-0.192181
O3	0.335100	1.421110	0.276880	0.344197	1.431563	0.282768
O4	0.146800	0.996350	0.691410	0.146920	0.994937	0.692181
O5	-0.164900	-0.921110	-0.223120	-0.155803	-0.931563	-0.217232
O6	-0.353200	-0.496350	0.191410	-0.353080	-0.494937	0.192181
O7	0.664900	-0.421110	0.723120	0.655803	-0.431563	0.717232
O8	0.853200	0.003650	0.308590	0.853080	0.005063	0.307819

Table S4: Atom coordinates relative to the unit cell lattice parameters for Ibuprofen.

Ibuprofen						
	Experimental data			Geometry optimization		
Element	x	y	z	x	y	z
H1	0.424700	0.566500	0.469300	0.430362	0.577576	0.466413
H2	0.294800	0.291900	0.260000	0.292676	0.297068	0.254691
H3	0.457100	0.040300	0.295600	0.452279	0.028157	0.289588
H4	0.429900	0.181000	0.171200	0.428550	0.185842	0.163891
H5	0.351400	0.017600	0.187500	0.345816	0.018838	0.172316
H6	0.181200	0.283000	0.394500	0.178747	0.285187	0.388751
H7	0.109700	0.126200	0.556000	0.105469	0.134266	0.554451
H8	0.345800	-0.197200	0.635900	0.343023	-0.206389	0.639071
H9	0.417500	-0.054400	0.473800	0.415374	-0.058317	0.474576

H10	0.227000	-0.210800	0.771400	0.226338	-0.215257	0.776774
H11	0.145500	-0.043100	0.764700	0.143857	-0.047023	0.768306
H12	0.051000	-0.193600	0.572100	0.045949	-0.195941	0.570276
H13	0.086500	-0.383700	0.824700	0.081630	-0.392357	0.833414
H14	0.010100	-0.215900	0.789500	0.005051	-0.213114	0.792832
H15	0.016300	-0.403100	0.702500	-0.019728	-0.412774	0.709537
H16	0.179900	-0.375000	0.515300	0.174216	-0.385629	0.511179
H17	0.079900	-0.496500	0.534500	0.074749	-0.507078	0.532805
H18	0.180600	-0.487100	0.653500	0.175641	-0.504705	0.658422
H19	0.424700	1.066500	0.030700	-0.430362	1.077576	0.033587
H20	0.294800	0.791900	0.240000	-0.292676	0.797068	0.245309
H21	0.457100	0.540300	0.204400	-0.452279	0.528157	0.210412
H22	0.429900	0.681000	0.328800	-0.428550	0.685842	0.336109
H23	0.351400	0.517600	0.312500	-0.345816	0.518838	0.327684
H24	0.181200	0.783000	0.105500	-0.178747	0.785187	0.111249
H25	0.109700	0.626200	-0.056000	-0.105469	0.634266	-0.054451
H26	0.345800	0.302800	-0.135900	-0.343023	0.293611	-0.139071
H27	0.417500	0.445600	0.026200	-0.415374	0.441683	0.025424
H28	0.227000	0.289200	-0.271400	-0.226338	0.284743	-0.276774
H29	0.145500	0.456900	-0.264700	-0.143857	0.452977	-0.268306
H30	0.051000	0.306400	-0.072100	-0.045949	0.304059	-0.070276
H31	0.086500	0.116300	-0.324700	-0.081630	0.107643	-0.333414
H32	0.010100	0.284100	-0.289500	-0.005051	0.286886	-0.292832
H33	0.016300	0.096900	-0.202500	0.019728	0.087226	-0.209537
H34	0.179900	0.125000	-0.015300	-0.174216	0.114371	-0.011179
H35	0.079900	0.003500	-0.034500	-0.074749	-0.007078	-0.032805
H36	0.180600	0.012900	-0.153500	-0.175641	-0.004705	-0.158422
H37	0.424700	-0.566500	-0.469300	-0.430362	-0.577576	-0.466413
H38	0.294800	-0.291900	-0.260000	-0.292676	-0.297068	-0.254691
H39	0.457100	-0.040300	-0.295600	-0.452279	-0.028157	-0.289588
H40	0.429900	-0.181000	-0.171200	-0.428550	-0.185842	-0.163891
H41	0.351400	-0.017600	-0.187500	-0.345816	-0.018838	-0.172316
H42	0.181200	-0.283000	-0.394500	-0.178747	-0.285187	-0.388751
H43	0.109700	-0.126200	-0.556000	-0.105469	-0.134266	-0.554451
H44	0.345800	0.197200	-0.635900	-0.343023	0.206389	-0.639071
H45	0.417500	0.054400	-0.473800	-0.415374	0.058317	-0.474576
H46	0.227000	0.210800	-0.771400	-0.226338	0.215257	-0.776774
H47	0.145500	0.043100	-0.764700	-0.143857	0.047023	-0.768306
H48	0.051000	0.193600	-0.572100	-0.045949	0.195941	-0.570276
H49	0.086500	0.383700	-0.824700	-0.081630	0.392357	-0.833414
H50	0.010100	0.215900	-0.789500	-0.005051	0.213114	-0.792832
H51	0.016300	0.403100	-0.702500	0.019728	0.412774	-0.709537
H52	0.179900	0.375000	-0.515300	-0.174216	0.385629	-0.511179
H53	0.079900	0.496500	-0.534500	-0.074749	0.507078	-0.532805
H54	0.180600	0.487100	-0.653500	-0.175641	0.504705	-0.658422
H55	0.424700	-0.066500	0.969300	0.430362	-0.077576	0.966413
H56	0.294800	0.208100	0.760000	0.292676	0.202932	0.754691
H57	0.457100	0.459700	0.795600	0.452279	0.471843	0.789588
H58	0.429900	0.319000	0.671200	0.428550	0.314158	0.663891
H59	0.351400	0.482400	0.687500	0.345816	0.481162	0.672316
H60	0.181200	0.217000	0.894500	0.178747	0.214813	0.888751
H61	0.109700	0.373800	1.056000	0.105469	0.365734	1.054451
H62	0.345800	0.697200	1.135900	0.343023	0.706389	1.139071

H63	0.417500	0.554400	0.973800	0.415374	0.558317	0.974576
H64	0.227000	0.710800	1.271400	0.226338	0.715257	1.276774
H65	0.145500	0.543100	1.264700	0.143857	0.547023	1.268306
H66	0.051000	0.693600	1.072100	0.045949	0.695941	1.070276
H67	0.086500	0.883700	1.324700	0.081630	0.892357	1.333414
H68	0.010100	0.715900	1.289500	0.005051	0.713114	1.292832
H69	0.016300	0.903100	1.202500	-0.019728	0.912774	1.209537
H70	0.179900	0.875000	1.015300	0.174216	0.885629	1.011179
H71	0.079900	0.996500	1.034500	0.074749	1.007078	1.032805
H72	0.180600	0.987100	1.153500	0.175641	1.004705	1.158422
C1	0.416200	0.348300	0.396200	0.415787	0.349601	0.393527
C2	0.350100	0.220700	0.321700	0.347252	0.221377	0.317381
C3	0.399600	0.108900	0.238300	0.396567	0.105800	0.231071
C4	0.303800	0.123800	0.420300	0.301779	0.123797	0.417623
C5	0.217300	0.172600	0.446600	0.214567	0.176134	0.442892
C6	0.176000	0.090200	0.538500	0.173333	0.090499	0.536353
C7	0.220300	-0.048400	0.606500	0.218023	-0.049822	0.608167
C8	0.307900	-0.096500	0.582100	0.305591	-0.100293	0.583127
C9	0.348600	-0.012700	0.487900	0.346947	-0.015082	0.489729
C10	0.174100	-0.138500	0.704500	0.172918	-0.143010	0.708369
C11	0.097000	-0.263900	0.646700	0.093325	-0.267957	0.647553
C12	0.041400	-0.321000	0.748600	0.036955	-0.324264	0.751856
C13	0.137300	-0.415200	0.583600	0.131664	-0.424564	0.583590
C14	0.416200	0.848300	0.103800	-0.415787	0.849601	0.106473
C15	0.350100	0.720700	0.178300	-0.347252	0.721377	0.182619
C16	0.399600	0.608900	0.261700	-0.396567	0.605800	0.268929
C17	0.303800	0.623800	0.079700	-0.301779	0.623797	0.082377
C18	0.217300	0.672600	0.053400	-0.214567	0.676134	0.057108
C19	0.176000	0.590200	-0.038500	-0.173333	0.590499	-0.036353
C20	0.220300	0.451600	-0.106500	-0.218023	0.450178	-0.108167
C21	0.307900	0.403500	-0.082100	-0.305591	0.399707	-0.083127
C22	0.348600	0.487300	0.012100	-0.346947	0.484918	0.010271
C23	0.174100	0.361500	-0.204500	-0.172918	0.356990	-0.208369
C24	0.097000	0.236100	-0.146700	-0.093325	0.232043	-0.147553
C25	0.041400	0.179000	-0.248600	-0.036955	0.175736	-0.251856
C26	0.137300	0.084800	-0.083600	-0.131664	0.075436	-0.083590
C27	0.416200	-0.348300	-0.396200	-0.415787	-0.349601	-0.393527
C28	0.350100	-0.220700	-0.321700	-0.347252	-0.221377	-0.317381
C29	0.399600	-0.108900	-0.238300	-0.396567	-0.105800	-0.231071
C30	0.303800	-0.123800	-0.420300	-0.301779	-0.123797	-0.417623
C31	0.217300	-0.172600	-0.446600	-0.214567	-0.176134	-0.442892
C32	0.176000	-0.090200	-0.538500	-0.173333	-0.090499	-0.536353
C33	0.220300	0.048400	-0.606500	-0.218023	0.049822	-0.608167
C34	0.307900	0.096500	-0.582100	-0.305591	0.100293	-0.583127
C35	0.348600	0.012700	-0.487900	-0.346947	0.015082	-0.489729
C36	0.174100	0.138500	-0.704500	-0.172918	0.143010	-0.708369
C37	0.097000	0.263900	-0.646700	-0.093325	0.267957	-0.647553
C38	0.041400	0.321000	-0.748600	-0.036955	0.324264	-0.751856
C39	0.137300	0.415200	-0.583600	-0.131664	0.424564	-0.583590
C40	0.416200	0.151700	0.896200	0.415787	0.150399	0.893527
C41	0.350100	0.279300	0.821700	0.347252	0.278623	0.817381
C42	0.399600	0.391100	0.738300	0.396567	0.394200	0.731071
C43	0.303800	0.376200	0.920300	0.301779	0.376203	0.917623

C44	0.217300	0.327400	0.946600	0.214567	0.323866	0.942892
C45	0.176000	0.409800	1.038500	0.173333	0.409501	1.036353
C46	0.220300	0.548400	1.106500	0.218023	0.549822	1.108167
C47	0.307900	0.596500	1.082100	0.305591	0.600293	1.083127
C48	0.348600	0.512700	0.987900	0.346947	0.515082	0.989729
C49	0.174100	0.638500	1.204500	0.172918	0.643010	1.208369
C50	0.097000	0.763900	1.146700	0.093325	0.767957	1.147553
C51	0.041400	0.821000	1.248600	0.036955	0.824264	1.251856
C52	0.137300	0.915200	1.083600	0.131664	0.924564	1.083590
O1	0.379200	0.496800	0.414800	0.380831	0.504282	0.407042
O2	0.496900	0.311700	0.437500	0.497266	0.307797	0.441631
O3	-0.379200	0.996800	0.085200	-0.380831	1.004282	0.092958
O4	-0.496900	0.811700	0.062500	-0.497266	0.807797	0.058369
O5	-0.379200	-0.496800	-0.414800	-0.380831	-0.504282	-0.407042
O6	-0.496900	-0.311700	-0.437500	-0.497266	-0.307797	-0.441631
O7	0.379200	0.003200	0.914800	0.380831	-0.004282	0.907042
O8	0.496900	0.188300	0.937500	0.497266	0.192203	0.941631
